# Characterization of Antiretroviral Therapy (ART) Adherence Phenotypes and Psychosocial Symptom Clusters Among Black/African American (AA) and Hispanic/Latine Adolescents and Young Adults (AYAs) with HIV in the Adherence Connection for Counseling, Education, and Support (ACCESS-II) Trial

**DOI:** 10.3390/tropicalmed10120332

**Published:** 2025-11-25

**Authors:** Ann-Margaret Navarra, Taehoon Ha, Eva Liang, Maurade Gormley, David R. Garcia, Jason Fletcher, Lloyd A. Goldsamt, Michael G. Rosenberg, Karin Hasegawa, Jie Yang

**Affiliations:** 1 School of Nursing, Stony Brook University, Stony Brook, NY 11794, USA; eva.liang.1@stonybrook.edu; 2 Biostatistical Consulting Core, School of Medicine, Stony Brook University, Stony Brook, NY 11794, USA; 3 School of Nursing, University of Connecticut, Storrs, CT 06268, USA; maurade.gormley@uconn.edu; 4 School of Nursing, University of Texas Rio Grande Valley, Rio Grande, TX 78582, USA; david.garcia07@utrgv.edu; 5 Rory Meyers College of Nursing, New York University, New York, NY 10016, USA; jason.fletcher@nyu.edu (J.F.); lg54@nyu.edu (L.A.G.); 6 Division of Infectious Diseases, Department of Pediatrics, Jacobi Medical Center, Bronx, NY 10461, USA; michael.rosenberg@nychhc.org; 7 Department of Applied Mathematics and Statistics, School of Engineering, Stony Brook University, Stony Brook, NY 11794, USA; karin.hasegawa@stonybrook.edu; 8 Department of Family, Population and Preventive Medicine, School of Medicine, Stony Brook University, Stony Brook, NY 11794, USA; jie.yang@stonybrookmedicine.edu

**Keywords:** HIV, phenotype, symptom cluster(s), chronic illness, self-management, adolescence, young adulthood

## Abstract

Antiretroviral therapy (ART) adherence behavior is heterogeneous among adolescents and young adults (AYAs) with HIV and influenced by individual and interpersonal psychosocial factors. The primary objective of this study is to characterize ART adherence phenotypes and psychosocial symptom clusters, as related to ART adherence and HIV viral load suppression. This analysis included 60 AYAs with HIV enrolled in an ART adherence support clinical trial. Self-reported ART adherence at baseline, 12-weeks, and 24-weeks was used to define four ART adherence phenotypes: consistently high adherence (YY), early-only adherence (YN), late-only adherence (NY), and consistently low adherence (NN). Symptom clusters were empirically derived from baseline psychosocial measures, including adherence self-efficacy, ART knowledge, HIV stigma, psychological distress (depression, anxiety, trauma), and social support. Linear mixed-effects models were used to examine psychosocial symptom outcomes at three timepoints (baseline, 12-weeks, and 24-weeks) and across groups with different adherence or viral load phenotypes. Using hierarchical clustering, four distinct clusters were identified, underscoring heterogeneity of psychosocial symptoms and patterns of ART and viral suppression. Findings from this analysis are among the first known characterizations of ART adherence phenotypes and psychosocial symptom clusters among AYAs with HIV. Heterogeneity in clusters underscores the need to examine other factors, such as resilience, not captured in the present study. Overall, these study findings contribute to improved understanding of the multi-level psychosocial influences of ART adherence and viral load suppression.

## 1. Introduction

In the United States (US), there were an estimated 32,000 new Human Immunodeficiency Virus (HIV) infections reported in 2022 [1]. Among the national goals set forth by the End the HIV Epidemic (EHE) initiative is the goal to reduce the number of new HIV infections to 9588 by 2025 and 3000 by 2030 [1]. A related EHE goal is for 95% of people living with HIV to achieve viral suppression, or have less than 200 copies of HIV per milliliter of blood [1]. Alarmingly, the current viral suppression rate among people with HIV with HIV is 65% [1], with even lower estimates observed among adolescents and young adults (AYAs) identifying as Black, African American (AA), and Hispanic/Latine [2]. Highly effective antiretroviral therapy (ART) is available, and a critical component of the EHE initiative, achieving viral suppression, requires consistently high levels (e.g., ≥80%) of ART adherence [3]. Promoting ART adherence self-management among AYAs with HIV presents significant challenges, as this cohort is estimated to miss more than half of their ART doses [4].

Adherence behaviors are diverse and impacted by multi-level socioecological factors, including individual and interpersonal psychosocial and structural determinants. Further, poorly defined mechanisms and unclear leverage points in current ART adherence strategies have perpetuated HIV healthcare inequities and unjust social processes [5,6]. To address this gap, identification of adherence phenotypes is an important step for advancing the development of directed and effective interventions. Although heterogeneous in its definition, a phenotype is a set of observable characteristics that are distinct between individuals [7]. Thus, the process of phenotyping involves the identification of a symptom cluster or pattern/frequency of a health behavior that is associated with one or more outcomes [8,9]. Similarly, symptom clusters are defined as the co-occurrence of two or more symptoms sharing similar underlying mechanisms and/or outcomes [10].

The characterization and reporting of behavioral phenotypes in the HIV behavioral science evidence base is relatively new [8,9]. Evidence supports that behavioral symptoms associated with HIV disease and related health outcomes are heterogeneous across subgroups of individuals. This heterogeneity is important, as variations in levels of ART exposure (time from initiation to discontinuation) are associated with HIV disease progression [11]. Our qualitative work showed that ART adherence challenges do not occur in isolation, but are often linked to a symptom cluster of high-risk behaviors, including behavioral/mental health challenges [12]. Thus, characterizing adherence phenotypes may help predict individual treatment responses [13], and measuring patterns of inter-participant variability can inform the development of more precise intervention strategies [11]. However, phenotyping has not been examined for cohorts of Black, AA, Hispanic, and Latine AYAs with HIV, who face numerous inequities driven by socioecological and structural barriers, underscoring the need for more precise and tailored interventions.

Several studies have laid the groundwork for understanding the potential of phenotyping to improve adherence behavior strategies and approaches. For example, in adults with HIV, patterns and frequency of multiple risk behaviors among men who have sex with men (MSM) were examined [8] in participants of the Pharmacokinetic and Clinical Observations in People over Fifty (POPPY) prospective cohort study [14]. The authors identified a “high risk” phenotype as having three of four key behaviors: current smoking, excess alcohol, sexually transmitted infection (past 12 months), and recent recreational drug use (past 6 months) [8]. Factors associated with this phenotype were younger age and depressive symptoms, with the high-risk phenotype being 3.32 times as likely to report < 90% adherence [8]. These findings support interventions focused on individuals with high-risk phenotypes to improve ART adherence behaviors.

Findings from three recent studies using participant data from the CNS HIV Anti-Retroviral Therapy Effects Research (CHARTER) cohort [15] highlight the multidimensional nature of patient characteristics. One study identified four novel biopsychosocial phenotypes related to neurocognitive symptoms among people with HIV, including cognition, depressive symptoms, and self-reported daily functioning. Factors contributing to the diversity of these biopsychosocial phenotypes included demographics, polypharmacy, substance use, and comorbidity [16]. In another CHARTER cohort study [15], the odds of impaired functioning were greater among adults with HIV and depressive symptoms and motor skill impairment [17]. The authors highlighted that modifiable factors, such as depression and adherence behaviors, can be addressed through interventions to promote optimal everyday functioning [17].

In children with perinatal HIV infection, identifying pediatric phenotypes based on clinical, virological, and immunological [18] characteristics has been described [18]. Specifically, three distinct HIV pediatric sub-phenotypes were identified in a cohort with similar long-term HIV virological suppression, with baseline HIV viral load (viral load, henceforth) as a differentiating prognostic factor [18]. The most favorable phenotype was characterized by higher immune reconstitution and slower disease progression, while the less favorable cluster exhibited greater senescence and a larger viral reservoir. Notably, adherence data was not described and was thus acknowledged as a study limitation [18]. This research highlights the importance of tailoring ART treatment regimens to individual clinical, virological, and immunological characteristics [18] and the need for a better understanding of how ART adherence influences these phenotypic distinctions.

Building on this evidence, the clinical trial described in this paper, Adherence Connection for Counseling, Education, and Support (ACCESS-II), tested the efficacy of a theory-based [12], synchronous [12,19,20,21], peer-led behavioral intervention compared to an asynchronous, web-based, active control arm [12,20,21]. We recruited AYAs with HIV (N = 60), ages 18–29 years, on prescribed ART with detectable viral loads (VL ≥ 20 copies/mL) from multiple sites in New York City (NYC). Guided by the SE model [19,22], we hypothesized that Black/AA and Hispanic/Latine AYAs with HIV randomized to ACCESS II would demonstrate significant improvements in 12- and 24-week self-reported ART adherence and HIV viral load (primary outcomes), as well as adherence self-efficacy, knowledge about ART, HIV stigma and disclosure concerns, social support, psychological distress (anxiety, depression, trauma), and substance use (secondary outcomes), when compared to the asynchronous web-based HIV ART adherence education active control arm [20].

The primary objectives of this analysis are to (1) characterize ART adherence phenotypes among Black/AA and Hispanic/Latine AYAs with HIV participants in the ACCESS II trial; (2) examine how psychosocial variables (adherence self-efficacy, ART knowledge, HIV stigma, psychological distress (depression, anxiety, and trauma), social support, and substance use) and symptom clusters relate to ART adherence and viral load suppression; and (3) describe the distribution of the ART adherence phenotypes across the primary symptom clusters.

## 2. Materials and Methods

### 2.1. Overview

ACCESS-II is a mobile health behavioral intervention, designed in response to the multi-level complexities and influences of ART adherence [12,20,21]. We recruited AYAs with HIV (N = 60), ages 18–29 years, prescribed ART, with a detectable viral load (VL ≥ 20 copies/mL), from multiple sites in New York City. The present analyses used ACCESS-II participant data collected at baseline, 12-week, and 24-week study visits between February 2022 and October 2023 [20]. A description of the dataset is provided below. Study procedures and related protocols were approved by the single and local institutional review board (IRB), which clearly delineated the protection of ACCESS-II participants. Additional information about this study is provided by ClinicalTrials.gov (Registration #: NCT04499781).

### 2.2. Dataset

This analysis included data from a total of 60 AYA with HIV participants enrolled in the ACCESS-II trial. Viral suppression status at 24-weeks was available for 51 participants; 9 participants had missing viral load data at 24-weeks. Adherence data was available for 49 participants at both the 12- and 24-week timepoints; adherence data was missing for 11 participants at one or both of these timepoints.

Thirteen SE variables were included, with the label *psychosocial* applied, as this analysis focused on individual and interpersonal influences of ART adherence (see summary of variables and study measures below). Among these 13 variables were the five domains of social support, as measured by the Patient-Reported Outcomes Measure Information System (PROMIS) Social Relationship scales—companionship, emotional support, informational support, instrumental support, and social isolation—which were included as pre-defined T-scores [23]. The remaining eight variables—ART knowledge (measured by the HIV Treatment Knowledge Scale), adherence self-efficacy (including the Adherence Self-Efficacy Beliefs and Outcome-Expectancies Subscales), Patient Health Questionnaire-9 (PHQ-9), General Anxiety Disorder-7 (GAD-7), Primary Care Post-Traumatic Stress Disorder-5 (PC-PTSD-5), and HIV stigma—were used as total scores. Additionally, scoring on the PC-PTSD-5 was treated as both a continuous and categorical variable; participants who ‘deny lifetime experience of trauma’ were assigned a score of 0 instead of missing.


**ACCESS II Study Variables and Measures**
Study VariableStudy Measures *Viral Load ^a^Quantitative Plasma HIV RNA (Viral Load) [24]ART Adherence ^a^3-Day Self-Reported Adherence [25,26]ART Knowledge ^b†^HIV Treatment Knowledge Scale [27]Adherence Self-Efficacy ^b†^HIV-Medication-Taking Self-Efficacy Scale (HIV MT SES) Adherence Self-Efficacy Beliefs Subscale Adherence Self-Efficacy Outcome-Expectancies Subscale [28]HIV Stigma ^b‡^The HIV Stigma Scale [29]Social Support (Five Domains) ^b‡^ATN iTech Short Measure of the PROMIS Social Relationship Scales—Companionship, Emotional Support, Informational Support, Instrumental Support, and Social Isolation [23,30,31]Psychological Distress ^b†^  Anxiety  Depression  TraumaGeneralized Anxiety Disorder (GAD-7) [32] Patient Health Questionnaire (PHQ-9) [33] Primary Care PTSD (PC-PTSD-5) [34]Substance Use ^b†^ATN Substance Use Measure [35]Note: PTSD = post-traumatic stress disorder; ATN = Adolescent Medicine Trials Network. ^a^ Primary study outcomes. ^b^ Secondary study outcomes. ^†^ Individual influence of ART behavior. ^‡^ Interpersonal influence of ART behavior. * Psychometric data described in narrative description for each measure.

### 2.3. Viral Suppression and ART Adherence Phenotypes—Definition

Participants were classified as either virally suppressed or not suppressed at week 24, using a cutoff of <20 copies/mL of HIV RNA. When analyzing viral load as a continuous variable, values below the level of detection were imputed as 10 copies/mL prior to log10 transformation. Participants missing viral load data at week 24 were excluded from the suppression-related analyses.

Viral suppression was defined using only 24-week viral load data due to a substantial lack of viral load measurements at 12-weeks, which limited the ability to construct reliable viral suppression trajectories. However, adherence trajectories were based on self-reported data at both 12 and 24-weeks. Of note, baseline adherence was excluded to reduce model complexity and minimize overfitting, given the limited sample size. These decisions were made to ensure conceptual consistency in the use of prospective timepoints, while balancing data availability and analytic rigor.

Adherence status was determined at both 12 and 24-weeks using a threshold of 0.95 (Yes (Y) > 0.95; No (N) ≤ 0.95). Based on these values, participants were grouped into one of four adherence trajectories: consistently high adherence (YY; adherent at both timepoints), early-only adherence (YN; adherent at 12-weeks only), late-only adherence (NY; adherent at 24-weeks only), and consistently low adherence (NN; non-adherent at both timepoints). **These trajectory categories are presented as the four ART adherence phenotypes in the analyses.**

### 2.4. Statistical Analysis

Sociodemographic, individual, and interpersonal characteristics were compared across groups such as those with different ART adherence phenotypes using Chi-square tests for categorical variables with *p*-values obtained using Monte Carlo simulation and Wilcoxon rank-sum tests or Kruskal–Wallis tests for continuous variables. Longitudinal psychosocial scores were compared using linear mixed-effects models (LMMs), including group, time, and group-by-time interaction as fixed effects to account for group-specific within-subject variability over time. The variance–covariance structure for repeated measures was guided by Akaike Information Criterion (AIC), selecting from possible covariance structures such as Autoregressive model of order one, Toeplitz, unstructured, and/or compound symmetry. For each LMM, potential covariates (e.g., age, sex at birth, ethnicity, log-transformed viral load, and CD4^+^ T-lymphocyte count) were considered. Variables with a *p*-value < 0.1 in univariate LMM models with the psychosocial score of interest were adjusted in the final LMM. Residual diagnosis was performed to confirm the model assumptions such as normality and linearity.

Hierarchical clustering was performed on the 13 standardized baseline psychosocial variables. Specifically, psychosocial symptom clusters were empirically derived from baseline psychosocial measures, including adherence self-efficacy, ART knowledge, HIV stigma, psychological distress (depression, anxiety, trauma), and social support. Euclidean distance was used as the dissimilarity metric, and Ward’s D2 method was applied as the linkage criterion. The optimal number of clusters was selected using the elbow method based on the linkage distance plot. These cluster labels were then used to classify participants into distinct psychosocial symptom profiles (i.e., clusters). Similar LMMs were used to evaluate longitudinal trends in viral load and ART adherence across these empirically derived subgroups (i.e., clusters).

For sensitivity analysis with respect to missing data, multiple imputations were performed using fully conditional specification, generating 42 imputed datasets to reflect the proportion of participants with incomplete observations. Logistic regression models were employed for imputing binary variables, while predictive mean matching was applied for continuous variables. For each imputed dataset, LMMs were fitted with fixed effects for group, time, and group-by-time interaction, adjusting for relevant baseline covariates including age, sex at birth, ethnicity, CD4^+^ T-lymphocyte count, and log-transformed viral load. The variance–covariance structure for repeated measures was selected within each dataset based on the Akaike Information Criterion, and the final structure was determined by majority rule across all imputations. Final estimates and confidence intervals were pooled across datasets using Rubin’s rules to ensure valid statistical inference under the imputation framework. All analysis was performed using SAS 9.4 (SAS Institute Inc., Cary, NC, USA), and the significance level was set at 0.05.

## 3. Results

Sociodemographic and clinical characteristics of participants were stratified by viral suppression status at 24-weeks are presented in Table 1. Among the 51 participants with available suppression data, 14 were categorized as suppressed (plasma viral load < 20 copies/mL) and 37 as not suppressed (plasma viral load ≥ 20 copies/mL). Approximately one third (35.7%) of participants achieving viral suppression were female at birth, compared to participants (62.16%) not achieving viral suppression. This difference was not statistically significant (*p*-value = 0.1270). In contrast, participants who achieved viral suppression had significantly lower baseline log10 plasma viral load (median = 1.96, IQR = 0.70) than those who did not achieve viral suppression (median = 3.91, IQR = 1.82, *p*-value of 0.0003).

**Figure A1:**
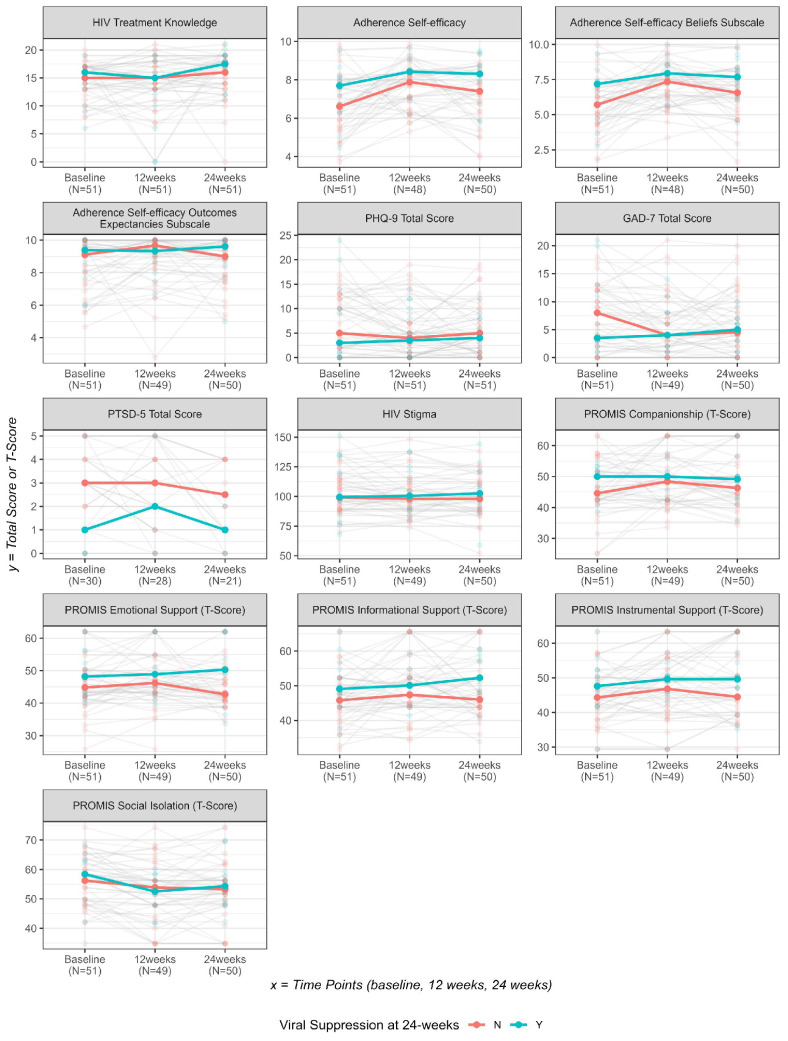
Total score and T-score plots by viral suppression.

Appendix A Figure A1 displays the longitudinal trajectories of these 13 psychosocial outcomes across the three timepoints by viral suppression group. This figure visually contextualizes the differences in psychosocial measures by viral suppression group, providing support for clustering. Individual-level patterns are shown in gray, while group-level medians for suppressed and not suppressed participants are overlaid to visualize trends and potential group differences over time (detailed descriptive statistics can be found in Appendix A, Table A1 and Table A2).

Findings from the linear mixed-effects models evaluating changes in psychosocial measures over time by 24-week viral suppression status are presented in Table 2. Scores on the HIV Treatment Knowledge Scale (score range = 0–21) showed a statistically significant interaction between suppression status and time (*p*-value = 0.0076), indicating that the pattern of change in knowledge scores differed between suppressed and non-suppressed groups over time. Of note, the estimated difference in knowledge scores between the suppressed and non-suppressed groups at baseline was −0.81 (95% CI: −3.12 to 1.49), and not statistically significant (*p*-value = 0.4824). At week 24, the direction of the difference reversed, with the suppressed group showing higher knowledge scores (estimate: 1.50, 95% CI: −0.60 to 3.61), although this individual timepoint was not statistically significant (*p*-value = 0.1577). The overall significant interaction suggests a divergent trend in knowledge scores across time between the two groups. For all other psychosocial variables, there was no evidence of differential change over time by viral suppression status (all *p*-values for interaction terms >0.05). In the sensitivity analysis using imputed datasets, none of the psychosocial measures demonstrated a significant group-by-time interaction, suggesting that psychosocial trajectories over time did not statistically differ across participants by suppression status (Table A3 in Appendix A).

### 3.1. Adherence Status at 12-Weeks and 24-Weeks (ART Adherence Phenotypes)

In Table 3, we present the sociodemographic and clinical characteristics across four ART adherence phenotypes. Participant characteristics (N = 49) are stratified by the **four ART adherence phenotypes**. As described, participants were grouped into one of four ART adherence phenotypes: consistently high adherence (YY, n = 22), early-only adherence (YN, n = 7), late-only adherence (NY, n = 8), and consistently low adherence (NN, n = 12). Among participants in the consistently high adherence group (YY, n = 22), 54.5% were male, while in the consistently low adherence group (NN, n = 12), 75.0% were female. Although these patterns suggest potential sex differences in these ART adherence phenotypes, such differences did not reach statistical significance (*p*-value = 0.3717).

In addition, no significant differences were observed across ART adherence phenotypes for age, race, ethnicity, HIV transmission route, or baseline HIV biomarkers. Similarly, no group differences were detected in lifetime or recent substance use patterns (all *p*-values > 0.05).

**Figure 1:**
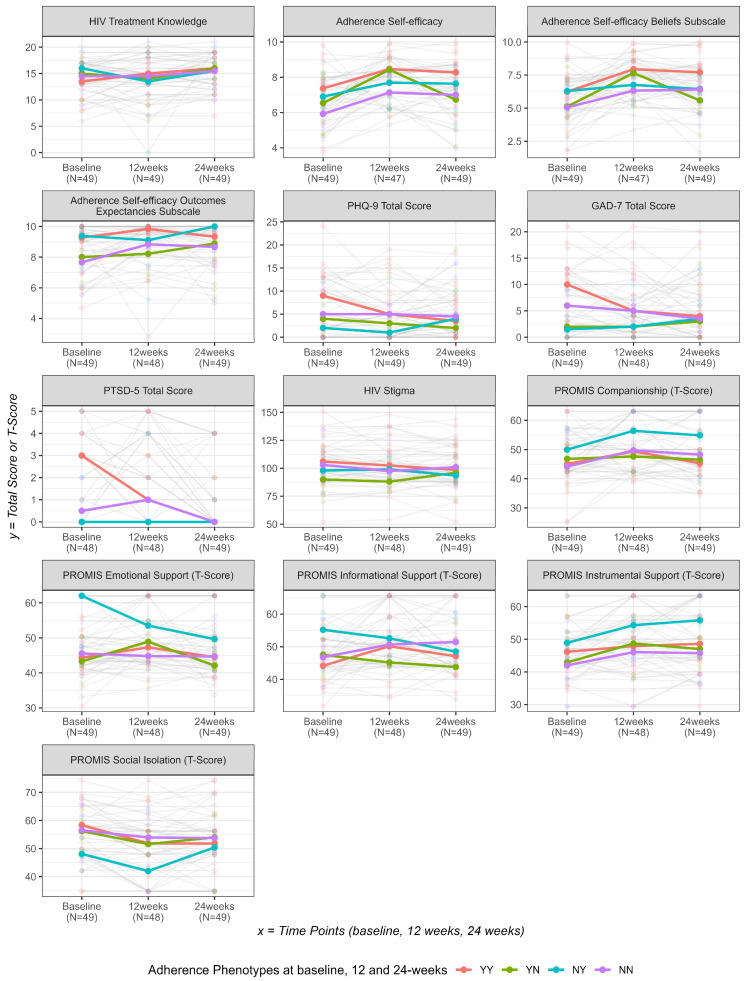
Total score and T-score plots by ART adherence phenotypes.

Figure 1 displays the longitudinal trajectories of 13 psychosocial outcomes across three timepoints by ART adherence phenotypes: consistently high adherence (YY), early-only adherence (YN), late-only adherence (NY), and consistently low adherence (NN). Additionally, more detailed descriptive statistics can be found in Appendix A, Table A4 and Table A5. This visualization highlights patterns and variability in psychosocial measures over time across different adherence behaviors.

Appendix A Table A6 summarizes results from linear mixed-effects models comparing longitudinal changes in psychosocial outcomes across the four ART adherence phenotype groups. In the model assessing adherence self-efficacy, the estimated difference at 24-weeks between the consistently adherent group (YY) and non-adherent group (NN) was 1.12 (95% CI: 0.20, 2.03; *p* = 0.0171), indicating a higher average adherence self-efficacy score in the YY group at that timepoint. Compared with the YY group, the early-only adherent group (YN) reported significantly lower adherence self-efficacy at 24-weeks (estimated difference = −1.53; 95% CI: −2.62, −0.44; *p* = 0.0065). However, this effect reflects a cross-sectional difference rather than a phenotype-by-time interaction and should be interpreted with caution in the absence of a significant global interaction term. Across all modeled outcomes, no statistically significant interaction effects were observed (all *p*-values for the interaction term of phenotype and time are >0.05), suggesting that changes in psychosocial measures over time did not differ statistically between ART adherence phenotype groups. Consistent conclusions were found from the sensitivity analysis using the multiple imputed datasets (Table A7 in the Appendix A).

### 3.2. Hierarchical Clustering of Psychosocial Factors and Linear Mixed-Effects Models by Cluster Labels

**Figure 2:**
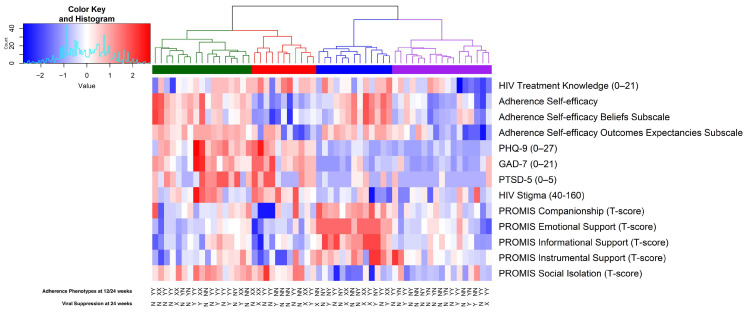
Hierarchically clustered heatmap of baseline psychosocial factors among AYAs with HIV.

**Figure A2:**
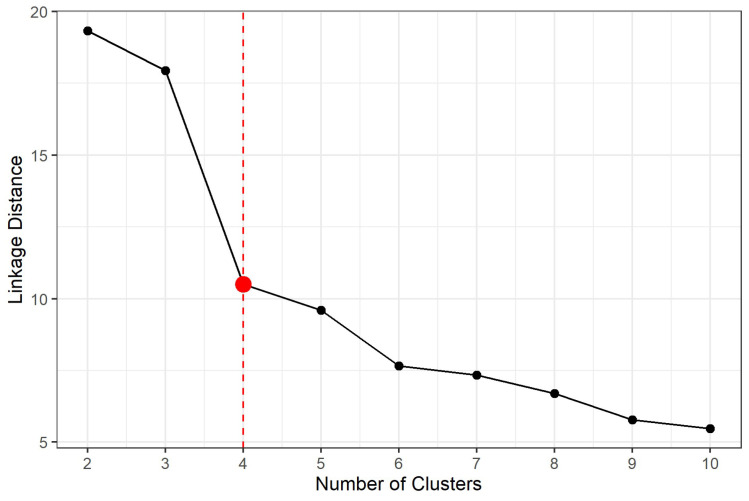
Elbow plot based on linkage distance in hierarchical clustering. The plot suggests an optimal cluster number of k = 4, where the decrease in linkage distance begins to level off. This value was subsequently used for hierarchical clustering and in the specification of cluster-level random effects in the linear mixed-effects model (LMM) analysis. Black dots represent linkage distances for each candidate number of clusters, the red dot highlights the selected optimal cluster number, and the vertical dashed red line indicates the corresponding cutoff.

The heatmap in Figure 2 displays hierarchical clustering of 13 psychosocial factors among 58 participants at *baseline,* with 2 participants excluded due to missing data. Each column represents a participant, and each row represents a z-scored psychosocial variable, including HIV treatment knowledge, adherence self-efficacy, psychological distress (PHQ-9, GAD-7, PTSD-5), HIV stigma, and social support (companionship, emotional, informational, instrumental, social isolation). Participants were grouped into four distinct psychosocial clusters (represented in Figure 2 as green, red, blue, and purple) using Ward’s D2 method on Euclidean distances. The optimal number of clusters was confirmed by the elbow method based on the linkage distance plot (see Appendix A Figure A2). On the *x*-axis, Y (Yes) indicates participants who achieved viral suppression at week 24 or who were categorized as adherent in the ART adherence phenotype at 12-weeks or 24-weeks. The label of N (No) indicates participants who did not achieve viral suppression at week 24 or who were categorized as non-adherent in the ART adherence phenotype at 12-weeks or 24-weeks. An ‘X’ symbol marks participants with missing data in the corresponding week.

**Cluster 1** (red, N = 17) is characterized by low psychological distress, including PHQ-9, GAD-7, and PC-PTSD-5, and relatively strong adherence self-efficacy. While this cluster shows consistently below-average scores in depression and anxiety domains, social support indicators (PROMIS companionship, emotional support, and informational support) exhibit a more mixed pattern without a clear directional trend. In contrast, **Cluster 2** (blue, N = 17) exhibited elevated psychological distress and HIV stigma, alongside low support. This cluster showed notably high scores on PHQ-9, GAD-7, PC-PTSD-5, and HIV stigma, with lower scores across all PROMIS support measures. **Cluster 3** (green, N = 11) demonstrated elevated scores on HIV treatment knowledge, psychological distress measures (PHQ-9, GAD-7, PC-PTSD-5), HIV stigma, and PROMIS social isolation. In contrast, this cluster had lower scores in adherence self-efficacy, on the Adherence Self-Efficacy Beliefs Subscale, and across all PROMIS support domains (companionship, emotional, informational, and instrumental support). Scores for the Adherence Self-Efficacy Outcome-Expectancies Subscale were more mixed, without a clear directional trend. Most z-scores in this cluster were below average, with little within-cluster variation. **Cluster 4** (purple, N = 13) demonstrated relatively high adherence self-efficacy scores, particularly across both belief and outcome domains. Treatment knowledge scores within this cluster were mixed, showing variability across participants. Psychological distress (PHQ-9, GAD-7, PC-PTSD-5) and HIV stigma measures were generally low. Among PROMIS social support indicators, scores for companionship, emotional support, informational support, and instrumental support were notably high. In contrast, social isolation scores were low, indicating strong perceived social connection and minimal feelings of isolation.

These four participant-level symptom cluster labels were used in subsequent linear mixed-effects models to assess differences in adherence and viral suppression trajectories. Table 4 presents sociodemographic and clinical characteristics of participants (N = 58) grouped by these four clusters. In comparing sociodemographic characteristics of participants by cluster labels, we found that the proportion of participants reporting lifetime marijuana use differed significantly across clusters (*p*-value = 0.0159). All participants in Cluster 2 (100%) reported prior marijuana use, compared to 65% in Cluster 1, 91% in Cluster 3, and 62% in Cluster 4. Similarly, differences were observed in lifetime tobacco use (*p*-value = 0.0337), with higher prevalence in Clusters 2 and 3 relative to Clusters 1 and 4. Although the distribution of HIV transmission routes approached significance (*p*-value = 0.0599), no statistically significant differences were detected across clusters for age, sex, ethnicity, alcohol use, or baseline biomarkers (*p*-values > 0.05).

Table 5 presents baseline values of individual psychosocial factors—including HIV treatment knowledge, adherence self-efficacy, depression (PHQ-9), anxiety (GAD-7), trauma exposure (PC-PTSD-5), and HIV stigma—across the four clusters. Statistically significant differences were observed in nearly all domains (*p* < 0.05), indicating meaningful heterogeneity in psychosocial functioning by cluster. For instance, Cluster 2 exhibited elevated scores for depression (PHQ-9 median = 12.00), anxiety (GAD-7 median = 9.00), trauma (PC-PTSD-5 median = 3.00), and HIV stigma (median = 105.00), in contrast to Cluster 1, which showed lower psychological distress across the same measures. Cluster 4 demonstrated high adherence self-efficacy with minimal psychological distress, while Cluster 3 showed low scores in adherence self-efficacy and high psychological distress and HIV stigma. In addition, we examined the interpersonal influence of social support (e.g., PROMIS T-scores) at baseline by cluster (see Table 6). Cluster 4 showed notably higher levels of emotional, informational, and instrumental support, as well as lower levels of social isolation compared to other clusters. Conversely, Clusters 2 and 3 exhibited the highest social isolation scores and lowest perceived supports (companionship, emotional, informational, instrumental).

**Figure 3:**
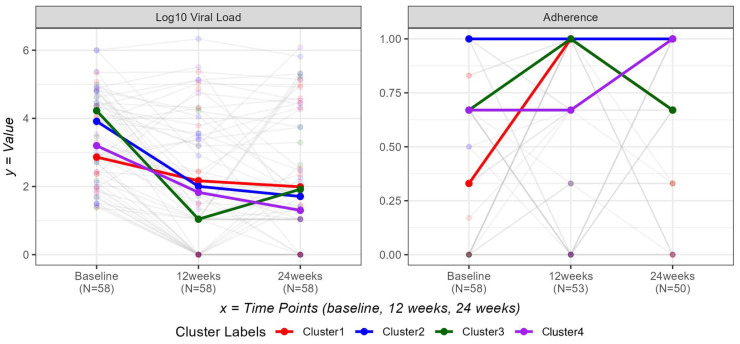
Viral load and ART adherence plots by cluster labels.

Figure 3 illustrates changes in log10 viral load and ART adherence across three timepoints (baseline, 12-weeks, 24-weeks) by clusters. All clusters showed a general decline in viral load over time, with Cluster 3 demonstrating the most pronounced reduction by week-12. In contrast, adherence levels varied more distinctly across clusters: Clusters 2 and 4 consistently reported high adherence, while Cluster 1 showed the largest improvement from baseline to 12-weeks, followed by stabilization.

Table 7 provides a summary of results from the linear mixed-effects models examining changes in log10-transformed viral load and ART adherence over time by baseline clusters (Clusters 1–4). For log10-transformed viral load, no statistically significant cluster-by-time interaction effects were observed (*p*-value = 0.5039). For adherence, while no significant overall cluster-by-time interaction effect was found (*p*-value = 0.1176), several pairwise differences emerged at specific timepoints. At baseline, Cluster 2 participants had significantly higher ART adherence scores than Cluster 1 (estimated difference = 0.32; 95% CI: 0.06, 0.57; *p*-value = 0.0157).

### 3.3. Summary of Clusters

Findings from our comparison of key demographic and psychosocial characteristics revealed significant differences in lifetime marijuana use (*p* = 0.0159) and tobacco use (*p* = 0.0337) across clusters, with Clusters 2 and 3 showing higher prevalence than Clusters 1 and 4. No significant differences were found for age, sex, ethnicity, alcohol use, or baseline HIV biomarkers.

Table 8 summarizes the primary characteristics of Clusters 1–4, including psychosocial profiles, ART adherence trajectories, and log_10_ viral load trends over time.

The adherence data includes N = 49 participants, and the cluster analysis was performed on N = 58 participants. To examine the overlap of ART adherence phenotype and clusters, a distinct dataset was compiled from participant data that appeared in either the adherence or cluster data (at least once). This resulted in a combined set of N = 59 unique participants. Table 9 shows the distribution of ART adherence phenotypes (YY, YN, NY, NN, or missing) across the four clusters and the unassigned participants for this combined dataset.

## 4. Discussion

Adolescence and young adulthood (AYA) encompass a period of significant physical, emotional, and neurocognitive flux. When coupled with a chronic and potentially fatal illness such as HIV, there are long-lasting implications for health and well-being. Maintaining life-long adherence to effective ART is key to optimizing viral suppression, immune competence, and individual and interpersonal psychosocial outcomes.

Findings from this analysis extend current evidence on ART adherence self-management behaviors among AYAs with HIV. We present foundational evidence for the characterization of four ART adherence phenotypes (YY, YN, NY, NN) and the identification of four distinct psychosocial symptom clusters (Clusters 1–4). We also describe how these four ART adherence phenotypes are distributed across symptom clusters.

More specifically, the four identified ART adherence phenotypes were characterized as follows: consistently high adherence (YY), early-only adherence (YN), late-only adherence (NY), and consistently low adherence (NN). A trend towards a binary pattern in ART adherence was observed, as more than half of the participants were either assigned to the consistently high (37%) or consistently low (20%) ART adherence phenotype. Among participants in the consistently high adherence phenotype (YY), more than half self-reported biologic sex as male. In contrast, three quarters of those in the consistently low-adherence phenotype (NN) self-reported female sex at birth. These patterns suggest potential biologic sex differences in these ART adherence phenotypes, although these differences were not statistically significant. We also did not observe statistically significant differences across the four ART adherence phenotypes for age, race, ethnicity, HIV transmission route, or baseline HIV biomarkers. This lack of observed racial or ethnic differences may reflect the relatively small and demographically homogeneous sample drawn from a single urban region, which likely limited statistical power and variability in race-related structural factors. Similarly, no group differences were detected in lifetime or recent substance use patterns.

We identified a statistically significant difference in adherence self-efficacy between the consistently high adherence (YY) and consistently low adherence phenotypes (NN) at 24-weeks. However, we interpreted this finding with caution, as no significant phenotype-by-time interaction was observed. This finding suggests a cross-sectional association rather than a sustained temporal effect. Moreover, no statistically significant differences or changes were detected over time across psychosocial measures, including adherence self-efficacy, depression, anxiety, trauma symptoms, HIV stigma, social support, and social isolation.

As described in the results, we identified four distinct psychosocial symptom clusters (Clusters 1–4). In brief, characteristics of AYAs with HIV in Cluster 1 included low psychological distress and relatively strong adherence self-efficacy. Social support indicators (PROMIS companionship, emotional support, and informational support) were mixed, without a clear directional trend. Thus, participants with positive psychosocial profiles may not initially present with high adherence yet may be particularly responsive to the intervention. In contrast, Cluster 2 AYAs with HIV exhibited elevated psychological distress and HIV stigma, alongside low social support. This may suggest the presence of unmeasured protective factors such as resilience or strong engagement with healthcare providers. Among AYAs with HIV in Cluster 3, elevated scores on HIV treatment knowledge, psychological distress measures (anxiety, depression, and trauma), HIV stigma, and PROMIS social isolation were observed. Our interpretation is that AYAs with HIV in this cluster may have less access to social support/resources and face more stressors (internal and or external). While they showed initial improvements in ART adherence and viral suppression, these gains were not sustained at 24-weeks. This may suggest the need for more tailored or sustained support among AYAs with HIV and this symptom cluster. Participants in Cluster 3 had lower scores in adherence self-efficacy, on the Adherence Self-Efficacy Beliefs Subscale, and across all PROMIS support domains (companionship, emotional, informational, and instrumental support). Descriptively, Cluster 3 was the only cluster that exhibited low adherence self-efficacy alongside high psychological distress and a decline in adherence and viral suppression after the initial 12-week improvements. This observation is exploratory and based on observed trajectories, and not formal statistical interaction testing. In Cluster 4, AYAs with HIV demonstrated relatively high adherence self-efficacy scores, mixed HIV treatment knowledge scores, and low psychological distress and HIV stigma. Scores for companionship, emotional, informational, and instrumental support were notably high, while social isolation was low, indicating strong perceived social connection and minimal feelings of social isolation.

### 4.1. Primary Adherence and Viral Load Outcomes by Cluster

As displayed in the results section (Figure 3), our primary outcomes (log10-transformed viral load and ART adherence) were examined by cluster across three timepoints (baseline, 12-weeks, 24-weeks). For log10-transformed viral load, there were no statistically significant cluster-by-time interaction effects (*p*-value = 0.5039). This finding suggests that differences in viral load trajectories across clusters were not statistically detected. However, these findings should be interpreted with caution, as limited power may have obscured meaningful differences.

Similarly, for ART adherence, the overall cluster-by-time interaction effect was not significant (*p*-value = 0.1176); however, several pairwise differences emerged at specific timepoints. Notably, at baseline, Cluster 2 participants had significantly higher adherence scores than Cluster 1 (estimated difference = 0.32; 95% CI: 0.06, 0.57; *p*-value = 0.0157). These findings reflect cross-sectional differences and should be interpreted cautiously, as they do not indicate differential trends over time.

Of note, AYA with HIV participants who achieved viral suppression had significantly lower baseline log10 plasma viral load measurement (median = 1.96, IQR = 0.70) as compared to those who did not (median = 3.91, IQR = 1.82), with a *p*-value of 0.0003, indicating a strong association between lower baseline viral load and subsequent viral suppression. These findings add to the current body of evidence underscoring the clinical relevance of examining viral load data and trends, as higher viral load values (i.e., range between 200 copies/mL and 999 copies/mL), even on one determination, increase the risk of subsequent virological failure (viral load ≥ 200 copies/mL) [36]. While programmatic definitions commonly apply a <200 copies/mL threshold, our analysis used the more stringent <20 copies/mL cutoff at week 24, consistent with prior ACCESS-II reports. Results should therefore be interpreted in light of this conservative definition. Moreover, a known, independent risk factor for virologic failure is low-level viremia (LLV) among adults—mean age 29 years [37]—defined as two or more consecutive viral load values of ≥50 to ≤999 copies/mL after 6months of ART [36]. Among adults with HIV, risk scores for LLV with baseline VL > 500,000 copies/mL and 100,000~500,000 copies/mL were significantly higher in comparison to those with baseline VL < 100,000 copies/mL [36], a finding consistent with data from other published studies [38,39]. As stated earlier, we did not observe differences by biologic sex. However, in studies including adult women with HIV, unsuppressed viremia is associated with virologic failure and increased risk for multimorbidity [40] and myocardial fibro-inflammation [41]. These biologic sex differences require further examination in AYAs with HIV.

To further evaluate the psychosocial clustering, we also examined the internal validity of the cluster solution. As shown in the Appendix A, an elbow plot (linkage distance versus number of clusters) is provided to illustrate the trade-off between additional clusters and explained variance. Given the modest sample size, more extensive resampling or quantitative indices such as silhouette or gap statistics may not be stable; we therefore limited our validation to this descriptive approach.

### 4.2. Adherence Phenotypes Across the Primary Symptom Cluster

We did not observe any consistent trends in the distribution of ART adherence phenotype trajectories (YY, YN, NY, NN, or missing) across the four symptom clusters and the unassigned participants for this combined dataset. This may be explained by the small numbers in these groups. However, a perplexing observation was the representation of the consistently high adherence phenotype (YY) in Cluster 2 (high psychological distress, low social support, and prior marijuana use). High adherence self-efficacy was also observed and may be a proxy for a related variable not measured in this analysis. Further examinations with a larger sample will be needed before arriving at any conclusions.

### 4.3. Substance Use by Cluster

Differences in lifetime marijuana use differed significantly across clusters (*p*-value = 0.0159). All participants in Cluster 2 (100%) reported prior marijuana use, compared to 65% in Cluster 1, 91% in Cluster 3, and 62% in Cluster 4. Similarly, differences were observed in lifetime tobacco use (*p*-value = 0.0337), with higher prevalence in Clusters 2 and 3 relative to Clusters 1 and 4. Although the distribution of HIV transmission routes approached significance (*p*-value = 0.0599), no statistically significant differences were detected across clusters for age, sex, ethnicity, alcohol use, or baseline biomarkers (all *p*-values > 0.05).

Marijuana use among people with HIV is high, with 77% of HIV-infected adults reporting lifetime marijuana use, compared to 44.5% of their uninfected counterparts [42]. Interestingly, findings from a recent systematic review showed little effect of marijuana use on HIV continuum of care outcomes, including ART adherence and viral load suppression [43]. However, the authors acknowledge the need for future research to explore the reasons for marijuana use and to develop interventions addressing misuse and/or comorbid psychiatric conditions [43]. We also acknowledge that participant data was collected in a state with legalization of marijuana, and as seen in other populations with HIV, this may have influenced patterns of use and potency [44].

### 4.4. Public Health Implications and Future Directions

More than half of US young adults, ages 18–34 years, are burdened with one or more chronic health conditions [45]. Phenotypic characterization is one approach that lends itself to promoting a wider understanding of health and the underlying mechanisms that influence health outcomes, thereby offering a pathway to develop tailored interventions for prevention and treatment [46]. Ongoing exposure to stressful psychosocial and environmental factors is a known contributor to health inequities in at-risk settings [47]. A biologically based disease-susceptible phenotype—activated by adverse social and environment factors—has been described [47]. In response, Bagby and colleagues underscore the need to better understand the biological mechanisms underlying health, and the role of protective variables and resilience factors within individuals and communities at risk.

Although the characterization of ART adherence phenotypes and psychosocial symptom clusters is relatively new for behavioral research among AYAs with HIV, valuable insights can be drawn from nurse-led studies in other chronic illnesses. For example, four distinct phenotypes of sleep, which were predictive of heart failure outcomes among adults, have been identified [48]. These findings provided important recommendations for tailoring interventions to specific phenotypes rather than a single characteristic [48]. The Nursing Science Precision Health Model is foundational to numerous studies that characterize phenotypes of lifestyle and environmental factors [49].

### 4.5. Limitations and Strengths

The observed lack of statistically significant meaningful differences in outcomes needs to be interpreted in the context of the small sample size and limited power. Additionally, baseline self-reported psychological data were used to compute and analyze psychosocial symptom clusters and were not validated by AYAs with HIV. Further, we acknowledge that cluster validation was limited to an elbow plot due to the small sample size and that the large number of comparisons increases the risk of type I error. While 95% confidence intervals were reported, formal multiple-testing corrections were not applied, given the exploratory nature of the analysis. Model assumptions were examined, and no violations were detected. Although participants were recruited from more than one site, the vast majority were enrolled at a single primary site. Therefore, we did not adjust for site in the models, and this should be considered a limitation, although confounding by site is unlikely given the dominance of one location. However, strengths of this analysis include the use of HIV biomarkers such as viral load, CD4^+^ T cell counts, and ART adherence data. These data, in addition to our psychosocial data, were collected at multiple timepoints, and imputation was used for missing values.

## 5. Conclusions

Findings from this analysis are among the first known characterizations of ART adherence phenotypes and psychosocial symptom clusters among AYAs with HIV participating in an adherence support clinical trial. Moreover, the ART adherence phenotypes and psychosocial symptom clusters identified in this study underscore the heterogeneity in treatment response. Some of these clusters maintained high ART adherence despite high levels of psychological distress or HIV stigma, while others with perceived protective factors, such as social support, continued to face ART adherence challenges. By illustrating heterogeneity in symptom clusters and treatment response to intervention, we highlight the importance of tailoring interventions to individual and interpersonal and psychosocial profiles, while underscoring the need to examine other factors, such as multi-level resilience and susceptibility [50].

Moving forward, application of a precision health model offers the potential for evidence-based care at individual, interpersonal (family, community), and population levels [51]. This research is largely underdeveloped in AYAs with HIV and could provide conceptual guidance for phenotypic characterization in other domains.

The EHE initiative was first announced in 2019, with public health leaders acknowledging that there is no single approach or strategy to accomplish its goals and that varied approaches would need to be implemented across inherently diverse communities [52]. We presently face a crucial and nonpartisan juncture in health and health policy; the stakes are high, and complacency poses the risk of halting and/or reversing progress made to date. “The EHE initiative has the potential to be one of the greatest domestic public health achievements in our nation’s history” [52] (p. 24). Understanding ART adherence phenotypes is an important step towards precision in the development of targeted, effective interventions [53,54]. Tailoring strategies and approaches to specific ART adherence phenotypes may help in achieving optimal viral suppression in comparison to interventions directed to one single characteristic (e.g., HIV treatment knowledge). There is substantial evidence to show the US public health relevance and economic cost benefits of effective behavioral interventions designed to eliminate health inequities and end the HIV epidemic [12,55].

## Figures and Tables

**Table 1:** Sociodemographic and clinical characteristics of participants (N = 51) stratified by viral suppression status at 24-weeks.

Variable	Level	Total (N = 51)	Suppressed (<20 Copies/mL) (n = 14)	Not Suppressed (≥20 Copies/mL) (n = 37)	*p*-Value *
Site	HIV Clinic	48 (94.12%)	12 (85.71%)	36 (97.30%)	0.1783
	HIV Non-Profit Community Health Plan	3 (5.88%)	2 (14.29%)	1 (2.70%)	
Age (Years)		26.00 ± 4.00	27.00 ± 4.00	25.00 ± 4.00	0.4963
Sex at Birth	Female	28 (54.90%)	5 (35.71%)	23 (62.16%)	0.1270
	Male	23 (45.10%)	9 (64.29%)	14 (37.84%)	
Race	American Indian/ Alaskan Native	1 (1.96%)	0 (0.00%)	1 (2.70%)	0.5540
	Black/African American	32 (62.75%)	11 (78.57%)	21 (56.76%)	
	White	2 (3.92%)	1 (7.14%)	1 (2.70%)	
	More Than One Race	6 (11.76%)	1 (7.14%)	5 (13.51%)	
	Unknown/Not Reported	10 (19.61%)	1 (7.14%)	9 (24.32%)	
Ethnicity	Hispanic/Latine	22 (43.14%)	4 (28.57%)	18 (48.65%)	0.2233
	Non-Hispanic/Latine	29 (56.86%)	10 (71.43%)	19 (51.35%)	
Substance Use					
Tobacco (Lifetime)	No	17 (33.33%)	6 (42.86%)	11 (29.73%)	0.5073
	Yes	34 (66.67%)	8 (57.14%)	26 (70.27%)	
Tobacco (Current Use: Past Three Months)	Never	11 (32.35%)	3 (37.50%)	8 (30.77%)	0.5919
	Once, Twice, or Monthly	10 (29.41%)	1 (12.50%)	9 (34.62%)	
	Daily or Almost Daily; Weekly	13 (38.24%)	4 (50.00%)	9 (34.62%)	
Alcohol (Lifetime)	No	7 (13.73%)	2 (14.29%)	5 (13.51%)	1.0000
	Yes	44 (86.27%)	12 (85.71%)	32 (86.49%)	
Alcohol (Current Use: Past Three Months)	Never	7 (15.91%)	1 (8.33%)	6 (18.75%)	0.1329
	Once, Twice, or Monthly	26 (59.09%)	5 (41.67%)	21 (65.62%)	
	Daily or Almost Daily; Weekly	11 (25.00%)	6 (50.00%)	5 (15.62%)	
Marijuana (Lifetime)	No	11 (21.57%)	2 (14.29%)	9 (24.32%)	0.4899
	Yes	40 (78.43%)	12 (85.71%)	28 (75.68%)	
Marijuana (Current Use: Past Three Months)	Never	3 (7.50%)	0 (0.00%)	3 (10.71%)	0.3778
	Once, Twice, or Monthly	11 (27.50%)	5 (41.67%)	6 (21.43%)	
	Daily or Almost Daily; Weekly	26 (65.00%)	7 (58.33%)	19 (67.86%)	
HIV Mode of Transmission	Horizontal	12 (23.53%)	5 (35.71%)	7 (18.92%)	0.2724
	Perinatal	39 (76.47%)	9 (64.29%)	30 (81.08%)	
HIV Biomarkers at Time of Consent Plasma Viral Load (copies/mL)		1580.00 ± 28,085.00	90.50 ± 187.00	8190.00 ± 43,631.00	**0.0003**
log10 Plasma Viral Load		3.20 ± 2.34	1.96 ± 0.70	3.91 ± 1.82	**0.0003**
CD4^+^ T-Lymphocyte Count (cells/mm^3^)		316.00 ± 433.00	389.50 ± 549.50	260.00 ± 404.00	0.0855

Note: There were no missing values for any variables except CD4^+^ T-lymphocyte count, which had 6 missing observations. * n (%); median ± IQR. For categorical variables, *p*-values were based on Chi-squared test with exact *p*-value from Monte Carlo simulation; for continuous variables, *p*-values were from Wilcoxon rank-sum test. *p*-values below 0.05 are highlighted in bold.

**Table 2:** Comparisons of different psychosocial measures over time across viral suppression phenotype based on linear mixed-effects models.

Outcome	Variable	Level	Estimated Difference Between Suppressed and Non-Suppressed Groups or Estimated Coefficient and 95% CI	*p*-Value *	*p*-Value **
HIV Treatment Knowledge (0–21)	Group × Time	Baseline	−0.81 (−3.12, 1.49)	0.4824	**0.0076**
		12-weeks	−1.11 (−4.05, 1.83)	0.4504	
		24-weeks	1.50 (−0.60, 3.61)	0.1577	
Adherence Self-Efficacy (0–26)	Group × Time	Baseline	0.13 (−0.78, 1.03)	0.7820	0.7502
		12-weeks	0.45 (−0.52, 1.41)	0.3602	
		24-weeks	0.52 (−0.39, 1.42)	0.2615	
	Sex at Birth	Female vs. Male	−0.24 (−0.82, 0.33)	0.3968	0.3968
Adherence Self-Efficacy Beliefs Subscale (0–17)	Group × Time	Baseline	0.06 (−1.07, 1.19)	0.9196	0.7617
		12-weeks	0.56 (−0.65, 1.76)	0.3618	
		24-weeks	0.49 (−0.64, 1.63)	0.3872	
	Sex at Birth	Female vs. Male	−0.72 (−1.43, −0.01)	**0.0459**	**0.0459**
Adherence Self-Efficacy Outcome-Expectancies Subscale (0–9)	Group × Time	Baseline	−0.04 (−1.09, 1.01)	0.9423	0.8652
		12-weeks	0.05 (−1.04, 1.13)	0.9334	
		24-weeks	0.26 (−0.79, 1.31)	0.6236	
	Age		0.07 (−0.04, 0.19)	0.2108	0.2108
PHQ-9 (0–27)	Group × Time	Baseline	−3.78 (−7.61, 0.05)	0.0531	0.1496
		12-weeks	−0.55 (−4.38, 3.28)	0.7750	
		24-weeks	−2.49 (−6.31, 1.34)	0.2001	
	CD4^+^ T-Lymphocyte Count		0.0058 (0.0010, 0.0106)	**0.0196**	**0.0196**
	Ethnicity	Hispanic/Latine vs. Non-Hispanic/Latine	2.46 (−0.21, 5.12)	0.0695	0.0695
GAD-7 (0–21)	Group × Time	Baseline	−3.71 (−7.62, 0.21)	0.0633	0.4944
		12-weeks	−2.00 (−6.00, 2.00)	0.3230	
		24-weeks	−2.62 (−6.54, 1.30)	0.1868	
	CD4^+^ T-Lymphocyte Count		0.0059 (0.0009, 0.0109)	**0.0218**	**0.0218**
	Ethnicity	Hispanic/Latine vs. Non-Hispanic/Latine	1.90 (−0.88, 4.67)	0.1749	0.1749
PC-PTSD-5 (0–5)	Group × Time	Baseline	−1.07 (−2.58, 0.45)	0.1612	0.4974
		12-weeks	−0.29 (−1.78, 1.21)	0.6999	
		24-weeks	−1.36 (−3.03, 0.31)	0.1081	
	Ethnicity	Hispanic/Latine vs. Non-Hispanic/Latine	−1.07 (−2.58, 0.45)	0.1618	0.1618
HIV Stigma (40–160)	Group × Time	Baseline	8.38 (−4.24, 20.99)	0.1903	0.3993
		12-weeks	4.60 (−8.13, 17.33)	0.4749	
		24-weeks	9.88 (−2.73, 22.50)	0.1229	
	Sex at Birth	Female vs. Male	5.90 (−4.48, 16.27)	0.2581	0.2581
PROMIS Companionship (T-score)	Group × Time	Baseline	2.01 (−3.50, 7.51)	0.4705	0.9724
		12-weeks	2.45 (−3.18, 8.09)	0.3892	
		24-weeks	2.60 (−2.90, 8.10)	0.3496	
PROMIS Emotional Support (T-score)	Group × Time	Baseline	2.47 (−2.79, 7.73)	0.3528	0.2345
		12-weeks	4.45 (−0.91, 9.81)	0.1026	
		24-weeks	6.45 (1.18, 11.71)	**0.0169**	
	Ethnicity	Hispanic/Latine vs. Non-Hispanic/Latine	0.26 (−3.74, 4.25)	0.8973	0.8973
PROMIS Informational Support (T-score)	Group × Time	Baseline	3.29 (−2.45, 9.03)	0.2579	0.6178
		12-weeks	0.95 (−4.92, 6.82)	0.7483	
		24-weeks	3.46 (−2.28, 9.20)	0.2342	
	Ethnicity	Hispanic/Latine vs. Non-Hispanic/Latine	−2.22 (−6.43, 1.99)	0.2940	0.2940
PROMIS Instrumental Support (T-score)	Group × Time	Baseline	1.73 (−3.67, 7.13)	0.5222	0.7650
		12-weeks	2.20 (−4.41, 8.80)	0.5064	
		24-weeks	0.30 (−6.63, 7.22)	0.9316	
PROMIS Social Isolation (T-score)	Group × Time	Baseline	2.62 (−3.94, 9.17)	0.4296	0.3165
		12-weeks	−1.04 (−7.68, 5.61)	0.7568	
		24-weeks	1.69 (−4.86, 8.24)	0.6093	

Note: Unstructured covariance was selected for HIV treatment knowledge [0–21] and instrumental support; autoregressive for adherence self-efficacy, Adherence Self-Efficacy Beliefs Subscale, and Companionship T-score; compound symmetry for Adherence Self-Efficacy Outcome-Expectancies Subscale, HIV Stigma Total, emotional support, informational support, and social isolation; Toeplitz for PHQ-9 Total Score and GAD-7 Total Score; and autoregressive for PC-PTSD-5 Total Score. * *p*-values are from Type III t-tests for pairwise comparisons at each timepoint. ** *p*-values are from Type III F-tests evaluating the group-by-time interaction in the model. *p*-values below 0.05 are highlighted in bold.

**Table 3:** Sociodemographic characteristics of participants (N = 49) by ART adherence phenotype at 12 and 24-weeks.

Variable	Level	Total (N = 49)	ART Adherence Phenotype at 12-Weeks and 24-Weeks				
			YY (n = 22)	YN (n = 7)	NY (n = 8)	NN (n = 12)	*p*-Value *
Site	HIV Clinic	47 (95.92%)	20 (90.91%)	7 (100.00%)	8 (100.00%)	12 (100.00%)	0.6338
	HIV Non-Profit Community Health Plan	2 (4.08%)	2 (9.09%)	0 (0.00%)	0 (0.00%)	0 (0.00%)	
Age (Years)		25.00 ± 4.00	26.00 ± 3.00	22.00 ± 4.00	25.50 ± 4.50	25.50 ± 6.00	0.1706
Sex at Birth	Female	27 (55.10%)	10 (45.45%)	3 (42.86%)	5 (62.50%)	9 (75.00%)	0.3717
	Male	22 (44.90%)	12 (54.55%)	4 (57.14%)	3 (37.50%)	3 (25.00%)	
Race	American Indian/ Alaskan Native	1 (2.04%)	0 (0.00%)	0 (0.00%)	0 (0.00%)	1 (8.33%)	0.7269
	Black/African American	32 (65.31%)	13 (59.09%)	6 (85.71%)	6 (75.00%)	7 (58.33%)	
	White	1 (2.04%)	0 (0.00%)	0 (0.00%)	0 (0.00%)	1 (8.33%)	
	More Than One Race	6 (12.24%)	3 (13.64%)	1 (14.29%)	1 (12.50%)	1 (8.33%)	
	Unknown/ Not Reported	9 (18.37%)	6 (27.27%)	0 (0.00%)	1 (12.50%)	2 (16.67%)	
Ethnicity	Hispanic/Latine	20 (40.82%)	11 (50.00%)	2 (28.57%)	2 (25.00%)	5 (41.67%)	0.6021
	Non-Hispanic/Latine	29 (59.18%)	11 (50.00%)	5 (71.43%)	6 (75.00%)	7 (58.33%)	
Substance Use							
Tobacco (Lifetime)	No	18 (36.73%)	6 (27.27%)	3 (42.86%)	3 (37.50%)	6 (50.00%)	0.6080
	Yes	31 (63.27%)	16 (72.73%)	4 (57.14%)	5 (62.50%)	6 (50.00%)	
Tobacco (Current Use: Past Three Months)	Never	11 (35.48%)	6 (37.50%)	2 (50.00%)	1 (20.00%)	2 (33.33%)	0.7083
	Once, Twice, or Monthly	10 (32.26%)	6 (37.50%)	2 (50.00%)	1 (20.00%)	1 (16.67%)	
	Daily or Almost Daily; Weekly	10 (32.26%)	4 (25.00%)	0 (0.00%)	3 (60.00%)	3 (50.00%)	
Alcohol (Lifetime)	No	7 (14.29%)	3 (13.64%)	1 (14.29%)	1 (12.50%)	2 (16.67%)	1.0000
	Yes	42 (85.71%)	19 (86.36%)	6 (85.71%)	7 (87.50%)	10 (83.33%)	
Alcohol (Current Use: Past Three Months)	Never	8 (19.05%)	4 (21.05%)	2 (33.33%)	0 (0.00%)	2 (20.00%)	0.9410
	Once, Twice, or Monthly	26 (61.90%)	11 (57.89%)	4 (66.67%)	5 (71.43%)	6 (60.00%)	
	Daily or Almost Daily; Weekly	8 (19.05%)	4 (21.05%)	0 (0.00%)	2 (28.57%)	2 (20.00%)	
Marijuana (Lifetime)	No	12 (24.49%)	5 (22.73%)	1 (14.29%)	3 (37.50%)	3 (25.00%)	0.8124
	Yes	37 (75.51%)	17 (77.27%)	6 (85.71%)	5 (62.50%)	9 (75.00%)	
Marijuana (Current Use: Past Three Months)	Never	3 (8.11%)	2 (11.76%)	1 (16.67%)	0 (0.00%)	0 (0.00%)	0.9576
	Once, Twice, or Monthly	9 (24.32%)	4 (23.53%)	1 (16.67%)	1 (20.00%)	3 (33.33%)	
	Daily or Almost Daily; Weekly	25 (67.57%)	11 (64.71%)	4 (66.67%)	4 (80.00%)	6 (66.67%)	
HIV Mode of Transmission	Horizontal	8 (16.33%)	4 (18.18%)	2 (28.57%)	0 (0.00%)	2 (16.67%)	0.4820
	Perinatal	41 (83.67%)	18 (81.82%)	5 (71.43%)	8 (100.00%)	10 (83.33%)	
HIV Biomarkers at Time of Consent Plasma Viral Load (copies/mL)		2870.00 ± 27,946.00	688.50 ± 71,237.00	7128.00 ± 11,572.00	19,822.50 ± 21,273.00	1843.50 ± 25,542.00	0.8616
log10 Plasma Viral Load		3.46 ± 2.02	2.84 ± 3.05	3.85 ± 1.23	4.29 ± 1.17	3.19 ± 1.99	0.8616
CD4^+^ T-Lymphocyte Count (cells/mm^3^)		284.00 ± 405.50	304.00 ± 310.00	351.00 ± 293.00	175.50 ± 290.00	320.00 ± 587.00	0.4138

Note: There were no missing values for any variables except CD4^+^ T-lymphocyte count, which had 5 missing observations. * n (%); median ± IQR. For categorical variables, *p*-values were based on Chi-squared test with exact *p*-values from Monte Carlo simulation; for continuous variables, *p*-values were from one-way ANOVA. *p*-values below 0.05 are highlighted in bold.

**Table 4:** Sociodemographic characteristics of participants (N = 58) by cluster labels (Clusters 1–4).

Variable	Level	Total (N = 58)	Cluster 1 (n = 17)	Cluster 2 (n = 17)	Cluster 3 (n = 11)	Cluster 4 (n = 13)	*p*-Value *
Site	HIV Clinic	53 (91.38%)	17 (100.00%)	16 (94.12%)	8 (72.73%)	12 (92.31%)	0.0879
	HIV Non-profit Community Health Plan	5 (8.62%)	0 (0.00%)	1 (5.88%)	3 (27.27%)	1 (7.69%)	
Age (Years)		25.00 ± 5.00	27.00 ± 4.00	24.00 ± 4.00	25.00 ± 5.00	25.00 ± 4.00	0.5953
Sex at Birth	Female	29 (50.00%)	10 (58.82%)	8 (47.06%)	5 (45.45%)	6 (46.15%)	0.9081
	Male	29 (50.00%)	7 (41.18%)	9 (52.94%)	6 (54.55%)	7 (53.85%)	
Race	American Indian/Alaskan Native	1 (1.72%)	0 (0.00%)	0 (0.00%)	0 (0.00%)	1 (7.69%)	0.1585
	Asian	1 (1.72%)	0 (0.00%)	0 (0.00%)	1 (9.10%)	0 (0.00%)	
	Black/African American	36 (62.07%)	11 (64.70%)	11 (64.71%)	4 (36.36%)	10 (76.93%)	
	White	2 (3.45%)	0 (0.00%)	1 (5.88%)	0 (0.00%)	1 (7.69%)	
	More Than One Race	6 (10.35%)	3 (17.65%)	0 (0.00%)	3 (27.27%)	0 (0.00%)	
	Unknown/ Not Reported	12 (20.69%)	3 (17.65%)	5 (29.41%)	3 (27.27%)	1 (7.69%)	
Ethnicity	Hispanic/Latine	25 (43.10%)	7 (41.18%)	8 (47.06%)	6 (54.55%)	4 (30.77%)	0.6843
	Non-Hispanic/Latine	33 (56.90%)	10 (58.82%)	9 (52.94%)	5 (45.45%)	9 (69.23%)	
Substance Use							
Tobacco (Lifetime)	No	19 (32.76%)	8 (47.06%)	2 (11.76%)	2 (18.18%)	7 (53.85%)	**0.0337**
	Yes	39 (67.24%)	9 (52.94%)	15 (88.24%)	9 (81.82%)	6 (46.15%)	
Tobacco (Current Use: Past Three Months)	Never	14 (35.90%)	4 (44.44%)	5 (33.33%)	3 (33.33%)	2 (33.33%)	0.3309
	Once, Twice, or Monthly	10 (25.64%)	3 (33.33%)	3 (20.00%)	2 (22.22%)	2 (33.33%)	
	Daily or Almost Daily; Weekly	15 (38.46%)	2 (22.22%)	7 (46.67%)	4 (44.44%)	2 (33.33%)	
Alcohol (Lifetime)	No	7 (12.07%)	3 (17.65%)	2 (11.76%)	1 (9.09%)	1 (7.69%)	0.9051
	Yes	51 (87.93%)	14 (82.35%)	15 (88.24%)	10 (90.91%)	12 (92.31%)	
Alcohol (Current Use: Past Three Months)	Never	10 (19.61%)	5 (35.71%)	1 (6.67%)	2 (20.00%)	2 (16.67%)	0.4446
	Once, Twice, or Monthly	31 (60.78%)	9 (64.29%)	11 (73.33%)	5 (50.00%)	6 (50.00%)	
	Daily or Almost Daily; Weekly	10 (19.61%)	0 (0.00%)	3 (20.00%)	3 (30.00%)	4 (33.33%)	
Marijuana (Lifetime)	No	12 (20.69%)	6 (35.29%)	0 (0.00%)	1 (9.09%)	5 (38.46%)	**0.0159**
	Yes	46 (79.31%)	11 (64.71%)	17 (100.00%)	10 (90.91%)	8 (61.54%)	
Marijuana (Current Use: Past Three Months)	Never	4 (8.70%)	2 (18.18%)	1 (5.88%)	0 (0.00%)	1 (12.50%)	0.1712
	Once, Twice, or Monthly	11 (23.91%)	2 (18.18%)	4 (23.53%)	3 (30.00%)	2 (25.00%)	
	Daily or Almost Daily; Weekly	31 (67.39%)	7 (63.64%)	12 (70.59%)	7 (70.00%)	5 (62.50%)	
HIV Mode of Transmission	Horizontal	15 (25.86%)	2 (11.76%)	5 (29.41%)	6 (54.55%)	2 (15.38%)	0.0599
	Perinatal	43 (74.14%)	15 (88.24%)	12 (70.59%)	5 (45.45%)	11 (84.62%)	
HIV Biomarkers at Time of Consent Plasma Viral Load (copies/mL)		2400.00 ± 29,643.00	728.00 ± 10,911.00	8190.00 ± 63,290.00	16,800.00 ± 44,150.00	1580.00 ± 23,292.00	0.7704
log10 Plasma Viral Load		3.37 ± 2.11	2.86 ± 1.68	3.91 ± 2.82	4.23 ± 2.47	3.20 ± 1.53	0.7704
CD4^+^ T-Lymphocyte Count (cells/mm^3^)		304.00 ± 460.00	237.00 ± 227.00	316.00 ± 366.00	431.00 ± 577.00	245.50 ± 545.00	0.7190

Note: There were no missing values for any variables except CD4^+^ T-lymphocyte count, which had 7 missing observations. * For categorical variables, *p*-values were based on Chi-squared test with exact *p*-value from Monte Carlo simulation; for continuous variables, *p*-values were from Wilcoxon rank-sum test. *p*-values below 0.05 are highlighted in bold.

**Table 5:** Baseline individual and interpersonal influences by time and cluster labels.

Measure	Level	Cluster 1 Baseline (n = 17)	Cluster 2 Baseline (n = 17)	Cluster 3 Baseline (n = 11)	Cluster 4 Baseline (n = 13)	*p*-Value *
HIV Treatment Knowledge (0–21)		13.00 ± 6.00	15.00 ± 3.00	17.00 ± 3.00	15.00 ± 4.00	**0.0286**
Adherence Self-Efficacy (0–26)		6.04 ± 1.35	7.92 ± 0.73	5.62 ± 1.19	8.12 ± 1.69	**<0.0001**
Adherence Self-Efficacy Beliefs Subscale (0–17)		5.00 ± 2.06	7.00 ± 1.12	4.29 ± 2.94	7.18 ± 2.41	**<0.0001**
Adherence Self-Efficacy Outcome-Expectancies Subscale (0–9)		7.33 ± 2.33	9.78 ± 0.44	7.67 ± 3.22	9.67 ± 0.89	**<0.0001**
PHQ-9 (0–27)		0.00 ± 2.00	12.00 ± 5.00	11.00 ± 5.00	1.00 ± 3.00	**<0.0001**
PHQ-9 Categories	None–Minimal (0–4)	16 (94.12%)	2 (11.76%)	0 (0.00%)	10 (76.92%)	**<0.0001**
	Mild (5–9)	1 (5.88%)	3 (17.65%)	3 (27.27%)	3 (23.08%)	
	Moderate (10–14)	0 (0.00%)	8 (47.06%)	6 (54.55%)	0 (0.00%)	
	Moderately Severe (15–19)	0 (0.00%)	2 (11.76%)	1 (9.09%)	0 (0.00%)	
	Severe (20–27)	0 (0.00%)	2 (11.76%)	1 (9.09%)	0 (0.00%)	
GAD-7 (0–21)		2.00 ± 4.00	9.00 ± 7.00	12.00 ± 6.00	2.00 ± 3.00	**<0.0001**
GAD-7 Categories	Minimal (0–4)	14 (82.35%)	2 (11.76%)	1 (9.09%)	11 (84.62%)	**<0.0001**
	Mild (5–9)	1 (5.88%)	7 (41.18%)	1 (9.09%)	1 (7.69%)	
	Moderate (10–14)	2 (11.76%)	6 (35.29%)	6 (54.55%)	1 (7.69%)	
	Severe (15–21)	0 (0.00%)	2 (11.76%)	3 (27.27%)	0 (0.00%)	
PC-PTSD-5 (0–5)		0.00 ± 0.00	3.00 ± 3.00	3.00 ± 5.00	0.00 ± 2.00	**0.0002**
PC-PTSD-5 Categories	Denies Lifetime Experience of Trauma	11 (64.71%)	3 (17.65%)	3 (27.27%)	6 (46.15%)	**0.0067**
	Reports Lifetime Experience of Trauma:					
	Reports No Trauma Symptoms	3 (17.65%)	0 (0.00%)	0 (0.00%)	2 (15.38%)	
	Reports 1 to 2 Trauma Symptoms	1 (5.88%)	3 (17.65%)	1 (9.09%)	3 (23.08%)	
	Reports ≥3 or More Trauma Symptoms	2 (11.76%)	11 (64.71%)	7 (63.64%)	2 (15.38%)	
HIV Stigma (40–160)		95.00 ± 18.00	105.00 ± 25.00	114.00 ± 26.00	88.00 ± 18.00	**0.0017**

* For categorical variables, *p*-values were based on Chi-squared test with exact *p*-values from Monte Carlo simulation; for continuous variables, *p*-values were from Kruskal–Wallis test. *p*-values below 0.05 are highlighted in bold.

**Table 6:** Interpersonal influences (T-score) by cluster labels.

Variable	Cluster 1 (n = 17)	Cluster 2 (n = 17)	Cluster 3 (n = 11)	Cluster 4 (n = 13)
	Baseline	Baseline	Baseline	Baseline
Companionship	45 (5.0)	47 (7.3)	41.1 (12.2)	55.4 (4.7)
Emotional Support	44.1 (6.6)	43.7 (4.9)	41.4 (7.8)	60.5 (2.9)
Informational Support	45.1 (5.1)	45.4 (7.0)	41.1 (7.4)	59.4 (5.9)
Instrumental Support	46.1 (6.2)	42.9 (6.7)	39.3 (9.3)	52.1 (7.6)
Social Isolation	51.8 (6.5)	61.3 (4.7)	61.3 (6.4)	46.5 (7.4)

**Table 7:** Comparisons of viral load and ART adherence over time across baseline psychosocial clusters based on linear mixed-effects models.

Outcome ^1^	Variable	Time	Level	Estimated Difference and 95% CI	*p*-Value *	*p*-Value **
log10 Viral Load	Cluster × Time	Baseline	Cluster1 vs. Cluster2	0.77 (−0.08, 1.62)	0.0749	0.5039
			Cluster3 vs. Cluster2	−0.10 (−1.08, 0.87)	0.8323	
			Cluster4 vs. Cluster2	0.36 (−0.54, 1.26)	0.4221	
		12-weeks	Cluster1 vs. Cluster2	0.35 (−1.02, 1.72)	0.6091	
			Cluster3 vs. Cluster2	0.58 (−1.00, 2.15)	0.4648	
			Cluster4 vs. Cluster2	−0.07 (−1.52, 1.38)	0.9188	
		24-weeks	Cluster1 vs. Cluster2	0.11 (−1.29, 1.52)	0.8704	
			Cluster3 vs. Cluster2	−0.12 (−1.74, 1.49)	0.8782	
			Cluster4 vs. Cluster2	0.53 (−0.95, 2.01)	0.4764	
	CD4			−0.0014 (−0.0025, −0.0003)	**0.0131**	**0.0131**
	Sex at Birth		Female vs. Male	0.56 (−0.09, 1.20)	0.0916	0.0916
Adherence	Cluster × Time	Baseline	Cluster1 vs. Cluster2	0.32 (0.06, 0.57)	**0.0157**	0.1176
			Cluster3 vs. Cluster2	0.2906 (0.0021, 0.5792)	**0.0484**	
			Cluster4 vs. Cluster2	0.17 (−0.10, 0.45)	0.2146	
		12-weeks	Cluster1 vs. Cluster2	−0.04 (−0.29, 0.22)	0.7773	
			Cluster3 vs. Cluster2	0.12 (−0.18, 0.43)	0.4226	
			Cluster4 vs. Cluster2	0.08 (−0.22, 0.38)	0.5919	
		24-weeks	Cluster1 vs. Cluster2	0.16 (−0.12, 0.43)	0.2558	
			Cluster3 vs. Cluster2	0.04 (−0.28, 0.36)	0.8135	
			Cluster4 vs. Cluster2	−0.18 (−0.48, 0.12)	0.2463	
	Sex at Birth		Female vs. Male	−0.1412 (−0.2816, −0.0009)	**0.0486**	**0.0486**

^1^ Log10 viral load was adjusted for sex at birth and CD4^+^ T-lymphocyte count, and ART adherence was adjusted for sex at birth. Unstructured covariance was selected for log10-transformed viral load, and autoregressive covariance was selected for ART adherence in the linear mixed-effects model. Cluster 2 was used as the reference cluster in both models. * *p*-values were calculated based on type 3 *t*-test from the linear mixed-effects models. ** *p*-values were calculated based on type 3 F-test from the linear mixed-effects models. *p*-values below 0.05 are highlighted in bold.

**Table 8:** Overview of Clusters 1–4.

Cluster	Psychological Distress/ HIV Stigma/Substance Use	ART Adherence and log10 Change in Viral Load
1 (n = 17)	Low psychological distress High adherence self-efficacy Mixed patterns of social support	Highest increase in ART adherence from baseline to 12-weeks Steady decline in viral load
2 (n = 17)	High psychological distress and HIV stigma Prior marijuana use (100%)	Consistently high ART adherence from baseline to 24-weeks Steady decline in viral load
3 (n = 11)	High psychological distress and HIV stigma High HIV treatment knowledge and social isolation Low adherence self-efficacy and social support	Increase in ART adherence (baseline to 12-weeks) followed by a decrease at 24-weeks Most pronounced decrease in viral load at 12-weeks, yet not sustained
4 (n = 13)	Low psychological distress High adherence self-efficacy Mixed patterns of social support	Increase in ART adherence (baseline to 24-weeks) Steady decline in viral load

**Table 9:** **ART** adherence phenotype by cluster.

Adherence Phenotype	Cluster 1	Cluster 2	Cluster 3	Cluster 4	Unassigned to Any Cluster	Total
YY	6 (35.3%)	8 (47.1%)	4 (36.4%)	3 (23.1%)	1 (100.0%)	22
YN	5 (29.4%)	2 (11.8%)	0 (0.0%)	0 (0.0%)	0 (0.0%)	7
NY	2 (11.8%)	1 (5.9%)	0 (0.0%)	5 (38.5%)	0 (0.0%)	8
NN	4 (23.5%)	2 (11.8%)	4 (36.4%)	2 (15.4%)	0 (0.0%)	12
Missing Adherence	0 (0.0%)	4 (23.5%)	3 (27.3%)	3 (23.1%)	0 (0.0%)	10
Total	17	17	11	13	1	59

## Data Availability

This article analyzed existing data from the ACCESS-II trial (NR019535). No new data were created or analyzed in this study. The data that support the findings of this study are available from the corresponding author upon reasonable request and approval from the IRB.

## Abbreviations

The following abbreviations are used in this manuscript:

ART	Antiretroviral Therapy
ATN	Adolescent Medicine Trials Network
AYAs	Adolescents and Young Adults
EHE	End the HIV Epidemic
HIV	Human Immunodeficiency Virus
LMM	Linear Mixed-Effects Model

## Appendix A

**Table A1:** Individual and interpersonal influences by time and suppression status at 24-weeks.

Measure	N Missing	Level	Suppressed (<20 Copies/mL) at 24-Weeks *			Not Suppressed (≥20 Copies/mL) at 24-Weeks *		
			Baseline (n = 14)	12-Weeks (n = 14)	24-Weeks (n = 14)	Baseline (n = 37)	12-Weeks (n = 37)	24-Weeks (n = 37)
HIV Treatment Knowledge (0–21)	0		16.00 ± 3.00	15.00 ± 8.00	17.50 ± 3.00	15.00 ± 4.00	15.00 ± 4.00	16.00 ± 5.00
Adherence Self-Efficacy (0–26)	4		7.69 ± 1.73	8.42 ± 1.92	8.31 ± 1.38	6.62 ± 2.08	7.88 ± 1.62	7.40 ± 1.94
Adherence Self-Efficacy Beliefs Subscale (0–17)	4		7.18 ± 1.65	7.94 ± 2.47	7.68 ± 2.06	5.71 ± 2.59	7.35 ± 1.88	6.56 ± 2.32
Adherence Self-efficacy Outcomes Expectancies Subscale (0–9)	3		9.39 ± 1.67	9.33 ± 1.22	9.61 ± 1.11	9.11 ± 2.44	9.67 ± 1.56	9.00 ± 2.28
PHQ-9 (0–27)	0		3.00 ± 10.00	3.50 ± 10.00	4.00 ± 6.00	5.00 ± 10.00	4.00 ± 6.00	5.00 ± 8.00
PHQ-9 Categories	0	None or Minimal (0–4)	7 (50.00%)	7 (50.00%)	9 (64.29%)	16 (43.24%)	20 (54.05%)	17 (45.95%)
		Mild (5–9)	2 (14.29%)	3 (21.43%)	4 (28.57%)	6 (16.22%)	11 (29.73%)	11 (29.73%)
		Moderate (10–14)	3 (21.43%)	4 (28.57%)	1 (7.14%)	12 (32.43%)	2 (5.41%)	5 (13.51%)
		Moderately Severe (15–19)	0 (0.00%)	0 (0.00%)	0 (0.00%)	3 (8.11%)	4 (10.81%)	4 (10.81%)
		Severe (20–27)	2 (14.29%)	0 (0.00%)	0 (0.00%)	0 (0.00%)	0 (0.00%)	0 (0.00%)
GAD-7 (0–21)	3		3.50 ± 9.00	4.00 ± 6.50	5.00 ± 6.00	8.00 ± 10.00	4.00 ± 7.00	4.50 ± 9.00
GAD-7 Categories	3	Minimal (0–4)	8 (57.14%)	7 (58.33%)	7 (50.00%)	15 (40.54%)	20 (54.05%)	18 (50.00%)
		Mild (5–9)	2 (14.29%)	3 (25.00%)	6 (42.86%)	8 (21.62%)	8 (21.62%)	8 (22.22%)
		Moderate (10–14)	2 (14.29%)	2 (16.67%)	1 (7.14%)	11 (29.73%)	6 (16.22%)	7 (19.44%)
		Severe (15–21)	2 (14.29%)	0 (0.00%)	0 (0.00%)	3 (8.11%)	3 (8.11%)	3 (8.33%)
PC-PTSD-5 (0–5)	4		0.00 ± 3.00	0.00 ± 2.50	0.00 ± 0.00	1.00 ± 3.50	0.00 ± 3.00	0.00 ± 2.00
PC-PTSD-5 Categories	4	Denies Lifetime Experience of Trauma	5 (35.71%)	5 (41.67%)	9 (64.29%)	15 (41.67%)	16 (43.24%)	20 (55.56%)
		Reports Lifetime Experience of Trauma:						
		Reports No Trauma Symptoms	3 (21.43%)	2 (16.67%)	2 (14.29%)	2 (5.56%)	3 (8.11%)	3 (8.33%)
		Reports 1–2 Trauma Symptoms	2 (14.29%)	2 (16.67%)	3 (21.43%)	5 (13.89%)	7 (18.92%)	5 (13.89%)
		Reports 3–5 Trauma Symptoms	4 (28.57%)	3 (25.00%)	0 (0.00%)	14 (38.89%)	11 (29.73%)	8 (22.22%)
HIV Stigma (40–160)	3		99.50 ± 50.00	100.50 ± 34.50	102.50 ± 29.00	99.00 ± 24.00	98.00 ± 19.00	98.00 ± 23.00

* n(%); median ± IQR.

**Table A2:** Interpersonal influences (T-score) by suppression status at 24-weeks.

Interpersonal Influences	N Missing	Suppressed (≤20 Copies/mL) at 24-Weeks			Not Suppressed (≥20 Copies/mL) at 24-Weeks		
		Baseline	12-Weeks	24-Weeks	Baseline	12-Weeks	24-Weeks
		M (SD)	M (SD)	M (SD)	M (SD)	M (SD)	M (SD)
Companionship	3	48.5 (5.0)	50.4 (6.0)	50.1 (8.6)	45.9 (8.9)	48.2 (8.1)	47.9 (8.2)
Emotional Support	3	49.1 (7.6)	50.1 (8.4)	50.7 (9.4)	45.7 (8.4)	46.8 (7.9)	45.3 (6.9)
Informational Support	3	49.9 (8.2)	51 (6.8)	53.5 (6.8)	46.5 (8.0)	49.8 (9.2)	48.6 (8.7)
Instrumental Support	3	47.4 (8.0)	48.9 (8.3)	50.1 (9.5)	44.3 (7.9)	47.0 (9.6)	48.3 (10.0)
Social Isolation	3	57.9 (7.1)	52 (6.2)	54.7 (7.9)	55.4 (8.9)	52.7 (10.7)	52.8 (10.3)

**Table A3:** Estimates and 95% CIs for outcome scores over time based on linear mixed-effects models by suppression status at 24-weeks using imputed datasets.

Outcome	Variable	Level	Estimated Difference Between Suppressed and Non-Suppressed Groups or Estimated Coefficient and 95% CI	Relative Efficiency	*p*-Value *	*p*-Value **
HIV Treatment Knowledge (0–21)	Group × Time	Baseline	−0.46 (−2.37, 1.45)	0.9973	0.6304	0.3836
		12-weeks	−2.24 (−6.38, 1.90)	0.9901	0.2777	
		24-weeks	0.92 (−4.59, 6.42)	0.9857	0.7312	
Adherence Self-Efficacy (0–26)	Group × Time	Baseline	0.37 (−0.56, 1.31)	0.9938	0.4257	0.7398
		12-weeks	0.31 (−0.63, 1.26)	0.9935	0.5069	
		24-weeks	0.67 (−0.20, 1.54)	0.9963	0.1276	
	Sex at Birth	Female vs. Male	−0.42 (−0.95, 0.11)	0.9989	0.1191	0.1191
Adherence Self-Efficacy Beliefs Subscale (0–17)	Group × Time	Baseline	0.46 (−0.72, 1.65)	0.9966	0.4359	0.7939
		12-weeks	0.45 (−0.54, 1.44)	0.9938	0.3586	
		24-weeks	0.79 (−0.27, 1.84)	0.9957	0.1400	
	Sex at Birth	Female vs. Male	−0.80 (−1.46, −0.15)	0.9986	0.0177	0.0177
Adherence Self-Efficacy Outcome-Expectancies Subscale (0–9)	Group × Time	Baseline	0.08 (−0.97, 1.12)	0.9936	0.8796	0.7949
		12-weeks	−0.09 (−1.21, 1.04)	0.9913	0.8749	
		24-weeks	0.33 (−0.60, 1.27)	0.9977	0.4734	
	Age		0.08 (−0.02, 0.17)	0.9991	0.0990	0.0990
PHQ-9 (0–27)	Group × Time	Baseline	−0.47 (−3.80, 2.87)	0.9973	0.7785	0.8493
		12-weeks	−0.65 (−4.07, 2.77)	0.9963	0.7037	
		24-weeks	−1.44 (−4.90, 2.02)	0.9959	0.4055	
	CD4		0.0037 (−0.0006, 0.0080)	0.9964	0.0927	0.0927
	Ethnicity	Hispanic/Latine vs. Non-Hispanic/Latine	2.73 (0.48, 4.99)	0.9995	0.0184	0.0184
GAD-7 (0–21)	Group × Time	Baseline	−0.74 (−4.08, 2.61)	0.9972	0.6590	0.7851
		12-weeks	−0.64 (−4.22, 2.95)	0.9947	0.7220	
		24-weeks	−1.95 (−5.66, 1.75)	0.9935	0.2923	
	CD4		0.0045 (−0.0000, 0.0090)	0.9956	0.0506	0.0506
	Ethnicity	Hispanic/Latine vs. Non-Hispanic/Latine	2.15 (−0.20, 4.51)	0.9981	0.0723	0.0723
PTSD-5 (0–5)	Group × Time	Baseline	−0.33 (−1.39, 0.72)	0.9978	0.5289	0.6041
		12-weeks	0.03 (−1.14, 1.20)	0.9941	0.9632	
		24-weeks	−0.59 (−1.70, 0.52)	0.9960	0.2923	
	Ethnicity	Hispanic/Latine vs. Non-Hispanic/Latine	0.46 (−0.28, 1.20)	0.9987	0.2213	0.2213
HIV Stigma (40–160)	Group × Time	Baseline	4.31 (−7.74, 16.36)	0.9969	0.4752	0.9970
		12-weeks	4.43 (−9.03, 17.89)	0.9930	0.5081	
		24-weeks	4.74 (−8.75, 18.24)	0.9929	0.4804	
	Sex at Birth	Female vs. Male	7.81 (−1.39, 17.00)	0.9987	0.0944	0.0944
PROMIS Companionship (T-score)	Group × Time	Baseline	2.66 (−2.73, 8.05)	0.9961	0.3250	0.9802
		12-weeks	2.05 (−4.31, 8.42)	0.9908	0.5155	
		24-weeks	2.15 (−3.67, 7.97)	0.9934	0.4585	
PROMIS Emotional Support (T-score)	Group × Time	Baseline	2.84 (−2.88, 8.57)	0.9940	0.3213	0.8018
		12-weeks	2.78 (−2.99, 8.56)	0.9937	0.3349	
		24-weeks	4.52 (−1.32, 10.35)	0.9934	0.1252	
	Ethnicity	Hispanic/Latine vs. Non-Hispanic/Latine	−2.47 (−6.36, 1.43)	0.9982	0.2091	0.2091
PROMIS Informational Support (T-score)	Group × Time	Baseline	2.41 (−3.22, 8.04)	0.9956	0.3925	0.5962
		12-weeks	0.41 (−6.04, 6.86)	0.9913	0.8977	
		24-weeks	3.79 (−2.15, 9.73)	0.9937	0.2045	
	Ethnicity	Hispanic/Latine vs. Non-Hispanic/Latine	−3.44 (−7.23, 0.35)	0.9985	0.0739	0.0739
PROMIS Instrumental Support (T-score)	Group × Time	Baseline	2.88 (−2.99, 8.74)	0.9960	0.3284	0.9466
		12-weeks	2.39 (−4.52, 9.30)	0.9908	0.4854	
		24-weeks	1.80 (−4.05, 7.65)	0.9961	0.5386	
PROMIS Social Isolation (T-score)	Group × Time	Baseline	1.26 (−5.14, 7.66)	0.9954	0.6942	0.7796
		12-weeks	−0.86 (−7.74, 6.03)	0.9930	0.8025	
		24-weeks	0.81 (−6.41, 8.03)	0.9916	0.8212	

Note: Unstructured covariance was selected for HIV treatment knowledge [0–21], Self-Efficacy Beliefs Subscale, and PTSD-5 Total Score; autoregressive for adherence self-efficacy; compound symmetry for Self-Efficacy Outcome Expectations, HIV Stigma Total, emotional support, informational support, PHQ-9 Total Score, instrumental support, Companionship T-score, and social isolation; Toeplitz for GAD-7 Total Score. * *p*-values are from Type III *t*-tests for pairwise comparisons at each timepoint. ** *p*-values are from Type III F-tests evaluating the group-by-time interaction in the model.

**Table A4:** Individual and interpersonal influences by time and ART adherence phenotype at baseline, 12-weeks, and 24-weeks.

Variable	Level	N Missing	YY (n = 22) *			YN (n = 7) *			NY (n = 8) *			NN (n = 12) *		
			Baseline	12-Weeks	24-Weeks	Baseline	12-Weeks	24-Weeks	Baseline	12-Weeks	24-Weeks	Baseline	12-Weeks	24-Weeks
HIV Treatment Knowledge (0–21)		0	13.50 ± 6.00	15.00 ± 4.00	16.00 ± 5.00	15.00 ± 3.00	14.00 ± 10.00	16.00 ± 1.00	16.00 ± 2.50	13.50 ± 8.50	15.50 ± 5.50	14.50 ± 5.50	14.50 ± 5.00	15.50 ± 6.00
Adherence Self-Efficacy (0–26)		2	7.37 ± 1.88	8.46 ± 1.31	8.27 ± 1.31	6.54 ± 3.15	8.42 ± 2.35	6.73 ± 3.31	6.90 ± 1.25	7.69 ± 1.88	7.63 ± 1.52	5.92 ± 1.58	7.13 ± 1.46	7.00 ± 1.65
Adherence Self-Efficacy Beliefs Subscale (0–17)		2	6.24 ± 2.59	7.94 ± 1.47	7.71 ± 1.82	5.12 ± 3.18	7.65 ± 2.88	5.59 ± 2.94	6.29 ± 1.79	6.76 ± 1.88	6.44 ± 1.71	5.06 ± 2.24	6.32 ± 2.18	6.41 ± 2.62
Adherence Self-Efficacy Outcome-Expectancies Subscale (0–9)		1	9.28 ± 1.89	9.84 ± 0.56	9.33 ± 2.00	8.00 ± 2.89	8.22 ± 3.11	8.89 ± 3.22	9.39 ± 1.39	9.11 ± 2.56	10.00 ± 0.61	7.67 ± 3.44	8.84 ± 1.28	8.67 ± 2.22
PHQ-9 (0–27)		0	9.00 ± 12.00	5.00 ± 10.00	3.50 ± 8.00	4.00 ± 5.00	3.00 ± 3.00	2.00 ± 10.00	2.00 ± 5.00	1.00 ± 3.00	4.00 ± 3.00	5.00 ± 10.50	5.00 ± 6.50	4.50 ± 9.50
PHQ-9 Categories	None or Minimal (0–4)	0	10 (45.45%)	10 (45.45%)	11 (50.00%)	5 (71.43%)	5 (71.43%)	5 (71.43%)	5 (62.50%)	8 (100.00%)	5 (62.50%)	5 (41.67%)	5 (41.67%)	6 (50.00%)
	Mild (5–9)		2 (9.09%)	6 (27.27%)	8 (36.36%)	2 (28.57%)	1 (14.29%)	0 (0.00%)	2 (25.00%)	0 (0.00%)	2 (25.00%)	2 (16.67%)	6 (50.00%)	3 (25.00%)
	Moderate (10–14)		8 (36.36%)	2 (9.09%)	2 (9.09%)	0 (0.00%)	1 (14.29%)	1 (14.29%)	1 (12.50%)	0 (0.00%)	1 (12.50%)	4 (33.33%)	1 (8.33%)	1 (8.33%)
	Moderately Severe (15–19)		1 (4.55%)	4 (18.18%)	1 (4.55%)	0 (0.00%)	0 (0.00%)	1 (14.29%)	0 (0.00%)	0 (0.00%)	0 (0.00%)	1 (8.33%)	0 (0.00%)	2 (16.67%)
	Severe (20–27)		1 (4.55%)	0 (0.00%)	0 (0.00%)	0 (0.00%)	0 (0.00%)	0 (0.00%)	0 (0.00%)	0 (0.00%)	0 (0.00%)	0 (0.00%)	0 (0.00%)	0 (0.00%)
GAD-7 (0–21)		1	10.00 ± 10.00	5.00 ± 11.00	4.00 ± 7.00	2.00 ± 5.00	2.00 ± 2.00	3.00 ± 12.00	1.50 ± 6.50	2.00 ± 7.00	3.50 ± 6.50	6.00 ± 9.00	5.00 ± 3.00	3.50 ± 10.50
GAD-7 Categories	Minimal (0–4)		7 (31.82%)	11 (50.00%)	13 (59.09%)	5 (71.43%)	7 (100.00%)	4 (57.14%)	6 (75.00%)	5 (71.43%)	5 (62.50%)	6 (50.00%)	6 (50.00%)	6 (50.00%)
	Mild (5–9)	1	4 (18.18%)	3 (13.64%)	5 (22.73%)	1 (14.29%)	0 (0.00%)	1 (14.29%)	1 (12.50%)	1 (14.29%)	2 (25.00%)	3 (25.00%)	5 (41.67%)	2 (16.67%)
	Moderate Anxiety (10–14)		8 (36.36%)	5 (22.73%)	2 (9.09%)	1 (14.29%)	0 (0.00%)	2 (28.57%)	1 (12.50%)	1 (14.29%)	1 (12.50%)	3 (25.00%)	1 (8.33%)	3 (25.00%)
	Severe Anxiety (15–21)		3 (13.64%)	3 (13.64%)	2 (9.09%)	0 (0.00%)	0 (0.00%)	0 (0.00%)	0 (0.00%)	0 (0.00%)	0 (0.00%)	0 (0.00%)	0 (0.00%)	1 (8.33%)
PC-PTSD-5		2	3.00 ± 4.00	1.00 ± 3.00	0.00 ± 1.00	0.00 ± 0.00	0.00 ± 0.00	0.00 ± 1.00	0.00 ± 1.50	0.00 ± 2.00	0.00 ± 0.50	0.50 ± 2.50	1.00 ± 4.00	0.00 ± 1.50
PC-PTSD-5 Categories	Denies Lifetime Experience of Trauma	2	6 (28.57%)	5 (22.73%)	13 (59.09%)	6 (85.71%)	6 (85.71%)	5 (71.43%)	4 (50.00%)	3 (42.86%)	6 (75.00%)	5 (41.67%)	6 (50.00%)	5 (41.67%)
	Reports Lifetime Experience of Trauma:													
	Reports No Trauma Symptoms		1 (4.76%)	3 (13.64%)	2 (9.09%)	1 (14.29%)	0 (0.00%)	0 (0.00%)	1 (12.50%)	1 (14.29%)	0 (0.00%)	1 (8.33%)	0 (0.00%)	3 (25.00%)
	Reports 1–2 Trauma Symptoms		1 (4.76%)	6 (27.27%)	3 (13.64%)	0 (0.00%)	0 (0.00%)	2 (28.57%)	2 (25.00%)	2 (28.57%)	1 (12.50%)	3 (25.00%)	2 (16.67%)	2 (16.67%)
	Reports 3–5 Trauma Symptoms		13 (61.90%)	8 (36.36%)	4 (18.18%)	0 (0.00%)	1 (14.29%)	0 (0.00%)	1 (12.50%)	1 (14.29%)	1 (12.50%)	3 (25.00%)	4 (33.33%)	2 (16.67%)
HIV Stigma (40–160)		1	106.00 ± 30.00	102.50 ± 27.00	98.50 ± 31.00	90.00 ± 29.00	88.00 ± 19.00	96.00 ± 25.00	98.00 ± 14.50	99.00 ± 15.00	93.50 ± 11.00	103.00 ± 29.00	97.50 ± 26.50	101.00 ± 23.00

* n(%); Median +/− IQR.

**Table A5:** Interpersonal influences (T-score) by adherence status at 12-weeks and 24-weeks.

Variable	N Missing	YY (n = 22)			YN (n = 7)			NY (n = 8)			NN (n = 12)		
		Baseline	12-Weeks	24-Weeks	Baseline	12-Weeks	24-Weeks	Baseline	12-Weeks	24-Weeks	Baseline	12-Weeks	24-Weeks
Companionship	9	46.6 (10.7)	49.1 (8.1)	46.9 (9.0)	45.8 (5.4)	47.3 (5.3)	45.9 (3.3)	50.6 (5.0)	54.2 (9.5)	54.3 (8.8)	48 (7.5)	51.4 (8.1)	51 (8.7)
Emotional Support	9	46.2 (8.0)	49.1 (8.3)	47 (8.7)	43.9 (5.3)	47.1 (5.1)	44.1 (3.9)	55.9 (8.6)	52.8 (7.5)	51.9 (9.0)	46.2 (7.2)	48 (7.5)	47.7 (9.2)
Informational Support	9	47.1 (9.1)	51.1 (10.5)	49.5 (9.0)	46.7 (3.9)	47.9 (5.8)	47.3 (5.5)	54.6 (9.3)	53.2 (9.3)	52.2 (8.2)	47.2 (5.7)	52.8 (7.2)	51.5 (9.9)
Instrumental Support	9	46.9 (8.1)	49.3 (10.1)	49 (10.1)	46 (5.9)	47.9 (7.5)	45.9 (3.9)	48.7 (7.8)	52.1 (8.6)	53.4 (10.3)	42.3 (8.7)	46.3 (10.0)	48.3 (10.9)
Social Isolation	9	55.8 (10.4)	51.6 (11.7)	51.6 (11.6)	55.9 (5.3)	51.3 (5.2)	53.3 (9.2)	50.8 (6.5)	45.8 (10.9)	47.8 (8.5)	55.6 (9.3)	52.2 (10.1)	56.2 (9.1)

**Table A6:** Comparisons of different psychosocial measures over time across adherence phenotype based on linear mixed-effects models.

Outcome	Variable	Time	Level	Estimated Difference Between Adherence and Non-Adherence Groups or Estimated Coefficient and 95% CI	*p*-Value *	*p*-Value **
HIV Treatment Knowledge (0–21)	Group × Time	Baseline	NY vs. YY	2.47 (−0.23, 5.16)	0.0720	0.1101
		Baseline	YN vs. YY	1.59 (−1.24, 4.42)	0.2641	
		Baseline	NN vs. YY	−0.92 (−3.27, 1.42)	0.4311	
		12-weeks	NY vs. YY	−1.53 (−5.06, 1.99)	0.3854	
		12-weeks	YN vs. YY	−0.69 (−4.40, 3.01)	0.7074	
		12-weeks	NN vs. YY	−0.67 (−3.74, 2.39)	0.6597	
		24-weeks	NY vs. YY	0.40 (−2.30, 3.10)	0.7680	
		24-weeks	YN vs. YY	0.92 (−1.92, 3.75)	0.5191	
		24-weeks	NN vs. YY	0.06 (−2.29, 2.41)	0.9587	
Adherence Self-Efficacy (0–26)	Group × Time	Baseline	NY vs. YY	0.02 (−1.02, 1.06)	0.9654	0.6830
		Baseline	YN vs. YY	−0.73 (−1.83, 0.36)	0.1840	
		Baseline	NN vs. YY	0.97 (0.05, 1.88)	**0.0386**	
		12-weeks	NY vs. YY	−0.88 (−1.98, 0.22)	0.1141	
		12-weeks	YN vs. YY	−0.76 (−1.85, 0.34)	0.1729	
		12-weeks	NN vs. YY	0.89 (−0.03, 1.81)	0.0566	
		24-weeks	NY vs. YY	−0.60 (−1.64, 0.44)	0.2531	
		24-weeks	YN vs. YY	−1.53 (−2.62, −0.44)	**0.0065**	
		24-weeks	NN vs. YY	1.12 (0.20, 2.03)	**0.0171**	
	Sex at Birth		Female vs. Male	−0.32 (−0.84, 0.21)	0.2292	0.2292
Adherence Self-Efficacy Beliefs Subscale (0–17)	Group × Time	Baseline	NY vs. YY	−0.11 (−1.39, 1.18)	0.8687	0.5270
		Baseline	YN vs. YY	−0.69 (−2.03, 0.66)	0.3133	
		Baseline	NN vs. YY	0.85 (−0.28, 1.98)	0.1384	
		12-weeks	NY vs. YY	−1.10 (−2.46, 0.25)	0.1092	
		12-weeks	YN vs. YY	−0.80 (−2.15, 0.55)	0.2409	
		12-weeks	NN vs. YY	1.23 (0.09, 2.36)	**0.0343**	
		24-weeks	NY vs. YY	−1.11 (−2.39, 0.17)	0.0894	
		24-weeks	YN vs. YY	−2.03 (−3.37, −0.68)	**0.0036**	
		24-weeks	NN vs. YY	1.59 (0.47, 2.72)	**0.0061**	
	Sex at Birth		Female vs. Male	−0.78 (−1.42, −0.13)	**0.0191**	**0.0191**
Adherence Self-Efficacy Outcome-Expectancies Subscale (0–9)	Group × Time	Baseline	NY vs. YY	0.41 (−0.82, 1.64)	0.5068	0.4683
		Baseline	YN vs. YY	−0.71 (−2.04, 0.62)	0.2900	
		Baseline	NN vs. YY	1.00 (−0.07, 2.06)	0.0671	
		12-weeks	NY vs. YY	−0.26 (−1.54, 1.02)	0.6866	
		12-weeks	YN vs. YY	−0.57 (−1.90, 0.76)	0.3964	
		12-weeks	NN vs. YY	0.08 (−0.98, 1.15)	0.8747	
		24-weeks	NY vs. YY	0.50 (−0.73, 1.73)	0.4230	
		24-weeks	YN vs. YY	−0.48 (−1.82, 0.85)	0.4715	
		24-weeks	NN vs. YY	0.03 (−1.04, 1.10)	0.9555	
	Age			0.39 (−0.07, 0.15)	0.4664	0.4664
PHQ-9 (0–27)	Group × Time	Baseline	NY vs. YY	−2.48 (−6.99, 2.04)	0.2783	0.2434
		Baseline	YN vs. YY	−3.33 (−8.23, 1.56)	0.1793	
		Baseline	NN vs. YY	0.69 (−3.30, 4.69)	0.7300	
		12-weeks	NY vs. YY	−3.72 (−8.23, 0.79)	0.1050	
		12-weeks	YN vs. YY	−2.03 (−6.93, 2.86)	0.4108	
		12-weeks	NN vs. YY	2.25 (−1.75, 6.24)	0.2665	
		24-weeks	NY vs. YY	0.17 (−4.35, 4.68)	0.9409	
		24-weeks	YN vs. YY	1.06 (−3.83, 5.96)	0.6666	
		24-weeks	NN vs. YY	−1.02 (−5.01, 2.98)	0.6133	
	CD4			0.0040 (−0.0011, 0.0090)	0.1185	0.1185
	Ethnicity		Hispanic/Latine vs. Non-Hispanic/Latine	2.38 (−0.45, 5.22)	0.0967	0.0967
GAD-7 (0–21)	Group × Time	Baseline	NY vs. YY	−3.79 (−8.30, 0.73)	0.0988	0.0631
		Baseline	YN vs. YY	−5.67 (−10.56, −0.78)	**0.0236**	
		Baseline	NN vs. YY	2.49 (−1.50, 6.48)	0.2171	
		12-weeks	NY vs. YY	−2.70 (−7.30, 1.89)	0.2455	
		12-weeks	YN vs. YY	−5.39 (−10.27, −0.50)	**0.0311**	
		12-weeks	NN vs. YY	2.55 (−1.44, 6.53)	0.2076	
		24-weeks	NY vs. YY	−0.07 (−4.58, 4.44)	0.9757	
		24-weeks	YN vs. YY	1.17 (−3.71, 6.06)	0.6339	
		24-weeks	NN vs. YY	−0.35 (−4.34, 3.64)	0.8621	
	CD4^+^ T-Lymphocyte Count (cells/mm^3^)			0.0043 (−0.0008, 0.0095)	0.0958	0.0958
	Ethnicity		Hispanic/Latine vs. Non-Hispanic/Latine	1.94 (−0.94, 4.82)	0.1809	0.1809
PC-PTSD-5 (0–5)	Group × Time	Baseline	NY vs. YY	−1.36 (−3.30, 0.58)	0.1641	0.1168
		Baseline	YN vs. YY	−3.60 (−7.16, −0.03)	**0.0480**	
		Baseline	NN vs. YY	1.20 (−0.34, 2.74)	0.1214	
		12-weeks	NY vs. YY	−0.69 (−2.60, 1.22)	0.4661	
		12-weeks	YN vs. YY	2.10 (−1.24, 5.44)	0.2096	
		12-weeks	NN vs. YY	−0.99 (−2.57, 0.58)	0.2087	
		24-weeks	NY vs. YY	0.32 (−2.09, 2.74)	0.7867	
		24-weeks	YN vs. YY	0.08 (−2.62, 2.78)	0.9537	
		24-weeks	NN vs. YY	0.08 (−1.53, 1.69)	0.9210	
	Ethnicity		Hispanic/Latine vs. Non-Hispanic/Latine	0.50 (−0.54, 1.55)	0.3317	0.3317
HIV Stigma (40–160)	Group × Time	Baseline	NY vs. YY	−7.18 (−22.55, 8.18)	0.3555	0.2357
		Baseline	YN vs. YY	−13.30 (−29.35, 2.75)	0.1032	
		Baseline	NN vs. YY	−0.31 (−13.93, 13.30)	0.9636	
		12-weeks	NY vs. YY	−5.66 (−21.25, 9.93)	0.4727	
		12-weeks	YN vs. YY	−13.81 (−29.86, 2.24)	0.0909	
		12-weeks	NN vs. YY	0.55 (−13.07, 14.17)	0.9362	
		24-weeks	NY vs. YY	−6.50 (−21.87, 8.87)	0.4028	
		24-weeks	YN vs. YY	−2.05 (−18.10, 14.00)	0.8005	
		24-weeks	NN vs. YY	−3.00 (−16.61, 10.62)	0.6631	
	Sex at Birth		Female vs. Male	6.68 (−3.78, 17.15)	0.2049	0.2049
PROMIS Companionship (T-score)	Group × Time	Baseline	NY vs. YY	3.96 (−2.89, 10.81)	0.2534	0.9085
		Baseline	YN vs. YY	−0.87 (−8.07, 6.33)	0.8107	
		Baseline	NN vs. YY	−1.41 (−7.36, 4.54)	0.6379	
		12-weeks	NY vs. YY	5.26 (−1.82, 12.35)	0.1436	
		12-weeks	YN vs. YY	−1.82 (−9.01, 5.38)	0.6168	
		12-weeks	NN vs. YY	−2.25 (−8.20, 3.70)	0.4547	
		24-weeks	NY vs. YY	7.39 (0.55, 14.24)	**0.0346**	
		24-weeks	YN vs. YY	−1.02 (−8.21, 6.18)	0.7792	
		24-weeks	NN vs. YY	−4.07 (−10.02, 1.88)	0.1772	
PROMIS Emotional Support (T-score)	Group × Time	Baseline	NY vs. YY	9.34 (2.74, 15.93)	**0.0060**	0.5141
		Baseline	YN vs. YY	−2.61 (−9.51, 4.29)	0.4549	
		Baseline	NN vs. YY	0.09 (−5.58, 5.76)	0.9761	
		12-weeks	NY vs. YY	4.30 (−2.45, 11.06)	0.2088	
		12-weeks	YN vs. YY	−2.28 (−9.18, 4.62)	0.5135	
		12-weeks	NN vs. YY	1.18 (−4.49, 6.85)	0.6809	
		24-weeks	NY vs. YY	4.60 (−1.99, 11.19)	0.1691	
		24-weeks	YN vs. YY	−3.16 (−10.06, 3.74)	0.3647	
		24-weeks	NN vs. YY	−0.58 (−6.25, 5.09)	0.8392	
	Ethnicity		Hispanic/Latine vs. Non-Hispanic/Latine	−1.32 (−5.46, 2.82)	0.5239	0.5239
PROMIS Informational Support (T-score)	Group × Time	Baseline	NY vs. YY	6.64 (−0.40, 13.68)	0.0642	0.6001
		Baseline	YN vs. YY	−1.11 (−8.48, 6.27)	0.7661	
		Baseline	NN vs. YY	0.22 (−5.84, 6.29)	0.9414	
		12-weeks	NY vs. YY	2.25 (−5.00, 9.50)	0.5388	
		12-weeks	YN vs. YY	−3.95 (−11.33, 3.42)	0.2895	
		12-weeks	NN vs. YY	−1.38 (−7.44, 4.68)	0.6517	
		24-weeks	NY vs. YY	1.90 (−5.14, 8.94)	0.5931	
		24-weeks	YN vs. YY	−2.91 (−10.28, 4.47)	0.4356	
		24-weeks	NN vs. YY	−1.68 (−7.74, 4.38)	0.5830	
	Ethnicity		Hispanic/Latine vs. Non-Hispanic/Latine	−3.24 (−7.48, 1.00)	0.1313	0.1313
PROMIS Instrumental Support (T-score)	Group × Time	Baseline	NY vs. YY	1.80 (−5.68, 9.28)	0.6337	0.7493
		Baseline	YN vs. YY	−0.89 (−8.75, 6.97)	0.8234	
		Baseline	NN vs. YY	4.59 (−1.91, 11.09)	0.1640	
		12-weeks	NY vs. YY	3.73 (−3.96, 11.42)	0.3375	
		12-weeks	YN vs. YY	−1.32 (−9.18, 6.54)	0.7401	
		12-weeks	NN vs. YY	2.98 (−3.52, 9.48)	0.3655	
		24-weeks	NY vs. YY	4.48 (−3.00, 11.96)	0.2368	
		24-weeks	YN vs. YY	−3.05 (−10.92, 4.81)	0.4421	
		24-weeks	NN vs. YY	0.69 (−5.81, 7.19)	0.8339	
PROMIS Social Isolation (T-score)	Group × Time	Baseline	NY vs. YY	−5.04 (−13.21, 3.12)	0.2230	0.6384
		Baseline	YN vs. YY	0.11 (−8.47, 8.69)	0.9798	
		Baseline	NN vs. YY	0.15 (−6.94, 7.25)	0.9656	
		12-weeks	NY vs. YY	−5.10 (−13.42, 3.22)	0.2263	
		12-weeks	YN vs. YY	−0.32 (−8.90, 8.26)	0.9406	
		12-weeks	NN vs. YY	−0.57 (−7.67, 6.53)	0.8738	
		24-weeks	NY vs. YY	−3.80 (−11.96, 4.37)	0.3580	
		24-weeks	YN vs. YY	1.73 (−6.85, 10.31)	0.6903	
		24-weeks	NN vs. YY	−4.68 (−11.78, 2.41)	0.1932	

Note: An unstructured covariance structure was selected for HIV treatment knowledge (0–21); an autoregressive structure for adherence self-efficacy, Self-Efficacy Beliefs Subscale, and PROMIS Companionship T-score; compound symmetry for Self-Efficacy Outcome Expectancies, PHQ-9 Total Score, PTSD-5 Total Score, HIV Stigma Total, PROMIS emotional support, informational support, instrumental support, and social isolation; and a Toeplitz structure for GAD-7 Total Score. * *p*-values were calculated based on type 3 *t*-test from the linear mixed-effects models. ** *p*-values were calculated based on type 3 F-test from the linear mixed-effects models. *p*-values below 0.05 are highlighted in bold.

**Table A7:** Estimates and 95% CIs for outcome scores over time based on linear mixed-effects models by adherence at 12-weeks and 24-weeks using imputed datasets.

Outcome	Variable	Time	Level	Estimated Difference Between Adherence and Non-Adherence Groups or Estimated Coefficient and 95% CI	Relative Efficiency	*p*-Value *	*p*-Value **
HIV Treatment Knowledge (0–21)	Group × Time	Baseline	YN vs. YY	1.68 (−0.84, 4.20)	0.9978	0.1872	0.7901
		Baseline	NY vs. YY	2.16 (−0.25, 4.58)	0.9977	0.0783	
		Baseline	NN vs. YY	0.85 (−1.31, 3.01)	0.9973	0.4342	
		12-weeks	YN vs. YY	−0.37 (−5.63, 4.89)	0.9922	0.8871	
		12-weeks	NY vs. YY	−2.28 (−8.09, 3.54)	0.9886	0.4276	
		12-weeks	NN vs. YY	0.45 (−4.21, 5.12)	0.9909	0.8443	
		24-weeks	YN vs. YY	−0.97 (−8.09, 6.16)	0.9867	0.7801	
		24-weeks	NY vs. YY	−0.26 (−6.25, 5.72)	0.9894	0.9290	
		24-weeks	NN vs. YY	−0.12 (−5.76, 5.53)	0.9879	0.9664	
Adherence Self-Efficacy (0–26)	Group × Time	Baseline	YN vs. YY	−0.76 (−2.04, 0.53)	0.9927	0.2391	0.8735
		Baseline	NY vs. YY	0.14 (−1.05, 1.33)	0.9936	0.8114	
		Baseline	NN vs. YY	−0.78 (−1.84, 0.29)	0.9936	0.1467	
		12-weeks	YN vs. YY	−0.71 (−1.86, 0.43)	0.9966	0.2144	
		12-weeks	NY vs. YY	−0.70 (−1.78, 0.38)	0.9971	0.1977	
		12-weeks	NN vs. YY	−0.73 (−1.67, 0.21)	0.9981	0.1264	
		24-weeks	YN vs. YY	−1.27 (−2.38, −0.16)	0.9977	**0.0260**	
		24-weeks	NY vs. YY	−0.38 (−1.44, 0.69)	0.9977	0.4822	
		24-weeks	NN vs. YY	−0.82 (−1.78, 0.13)	0.9976	0.0875	
	Sex at Birth		Female vs. Male	−0.43 (−0.95, 0.09)	0.9990	0.1000	0.1000
Adherence Self-Efficacy Beliefs Subscale (0–17)	Group × Time	Baseline	YN vs. YY	−0.74 (−2.42, 0.93)	0.9951	0.3754	0.7770
		Baseline	NY vs. YY	0.09 (−1.48, 1.65)	0.9960	0.9104	
		Baseline	NN vs. YY	−0.71 (−2.11, 0.69)	0.9959	0.3120	
		12-weeks	YN vs. YY	−0.76 (−1.97, 0.45)	0.9965	0.2099	
		12-weeks	NY vs. YY	−0.74 (−1.90, 0.43)	0.9962	0.2102	
		12-weeks	NN vs. YY	−0.96 (−1.98, 0.07)	0.9969	0.0662	
		24-weeks	YN vs. YY	−1.73 (−3.05, −0.40)	0.9965	**0.0118**	
		24-weeks	NY vs. YY	−0.74 (−2.01, 0.52)	0.9967	0.2427	
		24-weeks	NN vs. YY	−1.22 (−2.35, −0.09)	0.9967	**0.0348**	
	Sex at Birth		Female vs. Male	−0.82 (−1.46, −0.18)	0.9986	**0.0134**	**0.0134**
Adherence Self-Efficacy Outcome-Expectancies Subscale (0–9)	Group × Time	Baseline	YN vs. YY	−0.63 (−2.07, 0.82)	0.9937	0.3858	0.6483
		Baseline	NY vs. YY	0.38 (−0.96, 1.72)	0.9941	0.5708	
		Baseline	NN vs. YY	−0.75 (−1.93, 0.42)	0.9944	0.2030	
		12-weeks	YN vs. YY	−0.45 (−1.81, 0.91)	0.9957	0.5072	
		12-weeks	NY vs. YY	−0.49 (−1.85, 0.87)	0.9936	0.4692	
		12-weeks	NN vs. YY	−0.13 (−1.24, 0.97)	0.9966	0.8110	
		24-weeks	YN vs. YY	−0.24 (−1.52, 1.04)	0.9982	0.7043	
		24-weeks	NY vs. YY	0.45 (−0.73, 1.64)	0.9988	0.4439	
		24-weeks	NN vs. YY	0.08 (−0.95, 1.12)	0.9992	0.8711	
	Age			0.07 (−0.03, 0.17)	0.9984	0.1849	0.1849
PHQ-9 (0–27)	Group × Time	Baseline	YN vs. YY	−2.18 (−7.37, 3.00)	0.9961	0.4001	0.5081
		Baseline	NY vs. YY	−2.33 (−7.35, 2.68)	0.9959	0.3534	
		Baseline	NN vs. YY	−0.19 (−4.44, 4.05)	0.9973	0.9278	
		12-weeks	YN vs. YY	−1.10 (−5.27, 3.07)	0.9963	0.5972	
		12-weeks	NY vs. YY	−3.74 (−7.69, 0.20)	0.9971	0.0625	
		12-weeks	NN vs. YY	−1.77 (−5.22, 1.68)	0.9970	0.3064	
		24-weeks	YN vs. YY	−0.11 (−4.50, 4.29)	0.9959	0.9610	
		24-weeks	NY vs. YY	−0.01 (−4.13, 4.12)	0.9970	0.9971	
		24-weeks	NN vs. YY	1.30 (−2.39, 5.00)	0.9959	0.4812	
	CD4^+^ T-Lymphocyte Count (cells/mm^3^)			0.0028 (−0.0014, 0.0069)	0.9979	0.1865	0.1865
	Ethnicity		Hispanic/Latine vs. Non-Hispanic/Latine	2.48 (0.21, 4.74)	0.9996	**0.0325**	**0.0325**
GAD-7 (0–21)	Group × Time	Baseline	YN vs. YY	−3.12 (−7.64, 1.39)	0.9967	0.1701	0.6150
		Baseline	NY vs. YY	−3.47 (−7.90, 0.95)	0.9961	0.1210	
		Baseline	NN vs. YY	−1.72 (−5.47, 2.04)	0.9973	0.3621	
		12-weeks	YN vs. YY	−3.81 (−8.56, 0.94)	0.9949	0.1124	
		12-weeks	NY vs. YY	−2.03 (−6.81, 2.75)	0.9935	0.3950	
		12-weeks	NN vs. YY	−2.06 (−5.94, 1.81)	0.9961	0.2888	
		24-weeks	YN vs. YY	0.41 (−4.35, 5.18)	0.9947	0.8612	
		24-weeks	NY vs. YY	0.01 (−4.53, 4.55)	0.9952	0.9972	
		24-weeks	NN vs. YY	0.68 (−3.21, 4.56)	0.9960	0.7267	
	CD4^+^ T-Lymphocyte Count (cells/mm^3^)			0.0039 (−0.0004, 0.0083)	0.9968	0.0750	0.0750
	Ethnicity		Hispanic/Latine vs. Non-Hispanic/Latine	2.08 (−0.33, 4.48)	0.9980	0.0889	0.0889
PC-PTSD-5 (0–5)	Group × Time	Baseline	YN vs. YY	−1.54 (−3.03, −0.04)	0.9963	**0.0442**	0.6577
		Baseline	NY vs. YY	−0.96 (−2.37, 0.44)	0.9970	0.1749	
		Baseline	NN vs. YY	−0.62 (−1.86, 0.61)	0.9971	0.3152	
		12-weeks	YN vs. YY	−1.12 (−2.69, 0.46)	0.9944	0.1599	
		12-weeks	NY vs. YY	−0.50 (−2.00, 1.00)	0.9947	0.5023	
		12-weeks	NN vs. YY	−0.35 (−1.61, 0.91)	0.9962	0.5805	
		24-weeks	YN vs. YY	−0.12 (−1.65, 1.41)	0.9954	0.8774	
		24-weeks	NY vs. YY	−0.19 (−1.59, 1.21)	0.9972	0.7846	
		24-weeks	NN vs. YY	0.02 (−1.21, 1.26)	0.9971	0.9710	
	Ethnicity		Hispanic/Latine vs. Non-Hispanic/Latine	0.50 (−0.24, 1.24)	0.9984	0.1806	0.1806
HIV Stigma (40–160)	Group × Time	Baseline	YN vs. YY	−7.58 (−24.04, 8.88)	0.9961	0.3586	0.8042
		Baseline	NY vs. YY	−4.13 (−19.81, 11.56)	0.9964	0.5989	
		Baseline	NN vs. YY	3.81 (−9.62, 17.25)	0.9982	0.5710	
		12-weeks	YN vs. YY	−11.55 (−28.42, 5.32)	0.9952	0.1744	
		12-weeks	NY vs. YY	−1.74 (−17.62, 14.13)	0.9960	0.8261	
		12-weeks	NN vs. YY	0.35 (−13.90, 14.59)	0.9959	0.9612	
		24-weeks	YN vs. YY	−1.10 (−18.90, 16.70)	0.9934	0.9010	
		24-weeks	NY vs. YY	−6.43 (−23.33, 10.47)	0.9938	0.4456	
		24-weeks	NN vs. YY	3.20 (−11.27, 17.68)	0.9953	0.6573	
	Sex at Birth		Female vs. Male	6.04 (−3.34, 15.42)	0.9986	0.2021	0.2021
PROMIS Companionship (T-score)	Group × Time	Baseline	YN vs. YY	−1.86 (−9.18, 5.47)	0.9962	0.6125	0.9950
		Baseline	NY vs. YY	3.04 (−3.85, 9.92)	0.9969	0.3793	
		Baseline	NN vs. YY	0.66 (−5.29, 6.60)	0.9978	0.8254	
		12-weeks	YN vs. YY	−1.80 (−9.19, 5.59)	0.9959	0.6259	
		12-weeks	NY vs. YY	3.31 (−3.67, 10.30)	0.9963	0.3447	
		12-weeks	NN vs. YY	1.72 (−4.35, 7.78)	0.9970	0.5718	
		24-weeks	YN vs. YY	−1.03 (−9.39, 7.34)	0.9919	0.8046	
		24-weeks	NY vs. YY	5.48 (−1.46, 12.42)	0.9965	0.1188	
		24-weeks	NN vs. YY	2.85 (−3.55, 9.25)	0.9950	0.3737	
PROMIS Emotional Support (T-score)	Group × Time	Baseline	YN vs. YY	−4.06 (−11.67, 3.54)	0.9943	0.2862	0.8809
		Baseline	NY vs. YY	7.01 (−0.21, 14.22)	0.9946	0.0567	
		Baseline	NN vs. YY	−1.32 (−7.25, 4.60)	0.9972	0.6557	
		12-weeks	YN vs. YY	−3.63 (−11.22, 3.96)	0.9944	0.3390	
		12-weeks	NY vs. YY	2.56 (−4.82, 9.94)	0.9939	0.4865	
		12-weeks	NN vs. YY	−1.56 (−7.48, 4.37)	0.9972	0.5992	
		24-weeks	YN vs. YY	−3.01 (−10.59, 4.57)	0.9944	0.4263	
		24-weeks	NY vs. YY	3.31 (−3.58, 10.20)	0.9963	0.3379	
		24-weeks	NN vs. YY	−0.10 (−6.28, 6.08)	0.9956	0.9742	
	Ethnicity		Hispanic/Latine vs. Non-Hispanic/Latine	−2.67 (−6.49, 1.15)	0.9982	0.1660	0.1660
PROMIS Informational Support (T-score)	Group × Time	Baseline	YN vs. YY	−2.61 (−10.08, 4.86)	0.9966	0.4849	0.8158
		Baseline	NY vs. YY	4.62 (−2.99, 12.24)	0.9943	0.2271	
		Baseline	NN vs. YY	−1.37 (−7.61, 4.87)	0.9969	0.6602	
		12-weeks	YN vs. YY	−3.87 (−11.32, 3.58)	0.9967	0.3014	
		12-weeks	NY vs. YY	0.05 (−7.80, 7.90)	0.9933	0.9893	
		12-weeks	NN vs. YY	1.17 (−5.17, 7.51)	0.9963	0.7113	
		24-weeks	YN vs. YY	−2.13 (−10.21, 5.95)	0.9938	0.5962	
		24-weeks	NY vs. YY	1.77 (−5.68, 9.22)	0.9951	0.6335	
		24-weeks	NN vs. YY	1.32 (−4.91, 7.55)	0.9970	0.6714	
	Ethnicity		Hispanic/Latine vs. Non-Hispanic/Latine	−3.72 (−7.56, 0.11)	0.9982	0.0566	0.0566
PROMIS Instrumental Support (T-score)	Group × Time	Baseline	YN vs. YY	−2.48 (−10.51, 5.55)	0.9958	0.5368	0.9742
		Baseline	NY vs. YY	0.87 (−6.66, 8.40)	0.9966	0.8167	
		Baseline	NN vs. YY	−4.23 (−10.73, 2.28)	0.9975	0.1975	
		12-weeks	YN vs. YY	−2.33 (−10.83, 6.18)	0.9939	0.5829	
		12-weeks	NY vs. YY	2.11 (−6.26, 10.47)	0.9929	0.6121	
		12-weeks	NN vs. YY	−2.81 (−9.51, 3.88)	0.9964	0.4022	
		24-weeks	YN vs. YY	−3.17 (−11.56, 5.22)	0.9943	0.4495	
		24-weeks	NY vs. YY	3.63 (−3.86, 11.12)	0.9968	0.3344	
		24-weeks	NN vs. YY	−1.23 (−7.85, 5.39)	0.9968	0.7099	
PROMIS Social Isolation (T-score)	Group × Time	Baseline	YN vs. YY	0.60 (−7.58, 8.77)	0.9979	0.8840	0.9741
		Baseline	NY vs. YY	−3.06 (−11.11, 4.98)	0.9968	0.4474	
		Baseline	NN vs. YY	1.30 (−5.51, 8.10)	0.9986	0.7039	
		12-weeks	YN vs. YY	0.52 (−8.18, 9.22)	0.9955	0.9051	
		12-weeks	NY vs. YY	−3.93 (−12.73, 4.88)	0.9936	0.3721	
		12-weeks	NN vs. YY	0.32 (−6.89, 7.54)	0.9963	0.9291	
		24-weeks	YN vs. YY	1.79 (−7.45, 11.03)	0.9935	0.6969	
		24-weeks	NY vs. YY	−2.28 (−10.96, 6.39)	0.9941	0.5975	
		24-weeks	NN vs. YY	4.12 (−3.44, 11.68)	0.9946	0.2775	

Note: An unstructured covariance structure was selected for HIV treatment knowledge (0–21) and PHQ-9 Total Score; an autoregressive structure for adherence self-efficacy, PTSD-5 Total Score, and PROMIS social isolation; compound symmetry for Self-Efficacy Outcome Expectations, PROMIS Companionship T-score, GAD-7 Total Score, HIV Stigma Total, PROMIS emotional support, informational support, and instrumental support; and a Toeplitz structure for Self-Efficacy Beliefs Subscale. * *p*-values are from Type III *t*-tests for pairwise comparisons at each timepoint, based on linear mixed-effects models performed on the imputed datasets. ** *p*-values are from joint Wald tests evaluating the group-by-time interaction in the model using combined estimates from multiple imputed datasets. *p*-values below 0.05 are highlighted in bold.
